# A genetic network that suppresses genome rearrangements in *Saccharomyces cerevisiae* and contains defects in cancers

**DOI:** 10.1038/ncomms11256

**Published:** 2016-04-13

**Authors:** Christopher D. Putnam, Anjana Srivatsan, Rahul V. Nene, Sandra L. Martinez, Sarah P. Clotfelter, Sara N. Bell, Steven B. Somach, Jorge E.S. de Souza, André F. Fonseca, Sandro J. de Souza, Richard D. Kolodner

**Affiliations:** 1 Ludwig Institute for Cancer Res., University of California School of Medicine, San Diego, 9500 Gilman Drive, La Jolla, California 92093-0669, USA; 2 Department of Medicine, University of California School of Medicine, San Diego, 9500 Gilman Drive, La Jolla, California 92093-0669, USA; 3 Instituto de Bioinformática e Biotecnologia, Natal 59082-180, Brazil; 4 Instituto Metrópole Digital, UFRN, Natal 59082-180, Brazil; 5 Instituto do Cérebro, UFRN, Natal 59082-180, Brazil; 6 Department of Cellular and Molecular Medicine, University of California School of Medicine, San Diego, 9500 Gilman Drive, La Jolla, California 92093-0669, USA; 7 Moores-UCSD Cancer Center, University of California School of Medicine, San Diego, 9500 Gilman Drive, La Jolla, California 92093-0669, USA; 8 Institute of Genomic Medicine, University of California School of Medicine, San Diego, 9500 Gilman Drive, La Jolla, California 92093-0669, USA

## Abstract

Gross chromosomal rearrangements (GCRs) play an important role in human diseases, including cancer. The identity of all Genome Instability Suppressing (GIS) genes is not currently known. Here multiple *Saccharomyces cerevisiae* GCR assays and query mutations were crossed into arrays of mutants to identify progeny with increased GCR rates. One hundred eighty two GIS genes were identified that suppressed GCR formation. Another 438 cooperatively acting GIS genes were identified that were not GIS genes, but suppressed the increased genome instability caused by individual query mutations. Analysis of TCGA data using the human genes predicted to act in GIS pathways revealed that a minimum of 93% of ovarian and 66% of colorectal cancer cases had defects affecting one or more predicted GIS gene. These defects included loss-of-function mutations, copy-number changes associated with reduced expression, and silencing. In contrast, acute myeloid leukaemia cases did not appear to have defects affecting the predicted GIS genes.

## Abstract (web summary)

Here, Richard Kolodner and colleagues use assays in *Saccharomyces cerevisiae* to identify 182 genetic modifiers of gross chromosomal rearrangements (GCRs). They also compared these Genome Instability Suppressing (GIS) genes and pathways in human cancer genome, and found many ovarian and colorectal cancer cases have alterations to GIS pathways.

## Graphical abstract

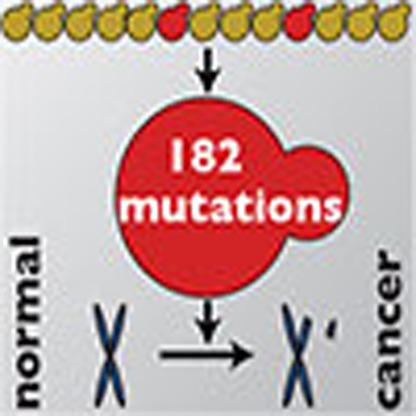

Genetic instability is seen in most cancers and is thought to play a critical role in the development and progression of tumours^1^. There are two general types of genetic instability seen in cancer^2^: the accumulation of large numbers of mutations and the accumulation of genome rearrangements such as translocations, copy-number changes and aneuploidy^2^,^3^. The study of cancer susceptibility syndromes like Fanconi Anemia and the *BRCA1*- and *BRCA2*-defective breast and ovarian cancer syndromes provided the first evidence for a causal link between defects causing increased genome rearrangements and the development of cancer^4^,^5^. However, this understanding is incomplete in part because most relevant studies have focused on a limited number of genes and the lack of genetic screens to identify Genome Instability Suppressing (GIS) genes in mammalian cells.

Due to the availability of quantitative genetic assays that can detect gross chromosomal rearrangements (GCRs), genetic studies in *Saccharomyces cerevisiae* have provided considerable information about the spontaneous formation of genome rearrangements^6^,^7^,^8^,^9^,^10^,^11^. The observed GCRs depend in part on the features of the specific GCR assay but include (1) terminal deletions healed by *de novo* telomere addition, (2) monocentric translocations, (3) interstitial deletions and (4) complex GCRs resulting from multiple cycles of rearrangement secondary to the formation of dicentric chromosomes by multiple processes^6^,^7^,^12^,^13^,^14^,^15^,^16^,^17^. Overall, the GCRs observed parallel to those being identified by whole-genome analysis in human diseases including cancer. In addition, GCR assays have been used to identify genes that prevent GCRs from occurring and genes that act in the formation of GCRs^6^,^7^,^8^,^9^,^10^,^15^,^18^,^19^,^20^,^21^,^22^,^23^,^24^,^25^,^26^,^27^.

Even in *S. cerevisiae*, our knowledge of GIS genes is incomplete. This is in part because most known GIS genes have been identified through limited candidate-gene approaches^6^,^7^,^8^,^10^,^15^,^18^,^19^,^28^. Only a small number of additional GIS genes have been identified in systematic screens^9^,^20^,^21^,^26^. Reasons for the limited success of these screens include: (1) the use of assays that were not specific for GCRs; (2) the low GCR rates detected in GCR assays are not well suited for large-scale genetic screens; (3) the use of only a single GCR assay; and (4) the lack of analysis of sufficient numbers of interacting mutations. Here we used a two-stage screen design in which an *in silico* approach was used to develop a highly enriched candidate gene list sorted into candidate pathways^29^ followed by an extensive genetic screen utilizing three different GCR assays and 43 query mutations to identify genes and interacting pairs of genes that act to suppress GCRs. Our results have provided a much more detailed picture of the genetic network that acts to prevent GCRs than previously available, and analysis of The Cancer Genome Atlas (TCGA) data^30^,^31^,^32^,^33^ has suggested that the genes in this network are potentially altered in a large proportion of ovarian and colorectal cancers but not in acute myeloid leukaemia.

## Results

### Design of the systematic genome instability screen

**Figure 1:**
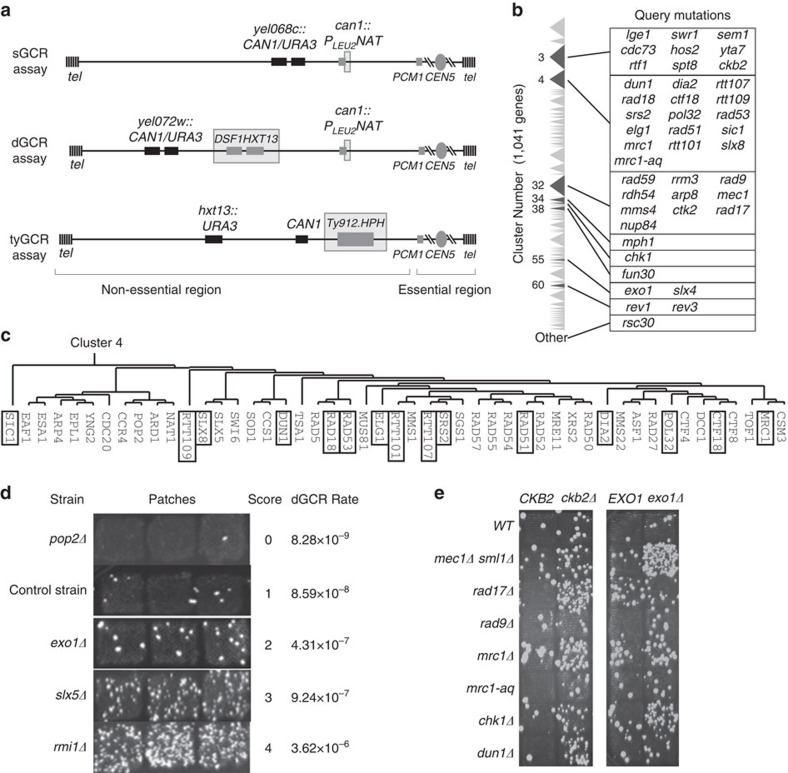
Details of the systematic screen. (**a**). The sGCR, dGCR, and tyGCR assays involve simultaneous selection against the *CAN1* and *URA3* genes inserted into the terminal non-essential region of the left arm of chromosome V. The GCR breakpoint region is the region between the telomeric *CAN1* and *URA3* genes and the most telomeric essential gene, *PCM1*. Homologies within the GCR breakpoint regions, including the ∼100 bp fragment of *YCLWdelta5* sequence introduced by *can1::P*_*LEU2*_*-NAT*, the *DSF1/HXT13* segmental duplication, and the inserted Ty912 element, are indicated with grey boxes. (**b**). The query mutations were primarily selected from the previously described gene clusters 3, 4, 32, 55, and 60 that were generated by clustering the candidate GCR-suppression genes by genetic interactions^29^. Clusters 3, 4, and 32 had the greatest number of GCR-suppressing genes. Triangles indicate the relative size of the cluster in terms of the number of genes, and the darker triangles are the clusters from which query mutations were selected. (**c**). Query mutations (indicated by the boxes) in non-essential genes in cluster 4 were selected to provide the greatest genetic diversity by picking 1 or 2 mutations from most sub-clusters. Query mutations were similarly selected from clusters 3 and 32. (**d**). The semi-quantitative scoring strategy assigns a number between 0 and 5 to each patch depending on the number of papillae (0: no papillae; 1: 1–5 papillae; 2: 6–15 papillae; 3: 16- a countable number of papillae (∼150–200); 4: papillae that were too many or too close together to count; 5 (not shown): a lawn of papillae covering the entire patch). For each strain, a minimum of 3 individual GCR patch scores were averaged to calculate the GCR strain score. Increases in the GCR strain score were paralleled by increases in GCR rates measured by the fluctuation method. (**e**). Patch tests documenting genetic interactions involving mutations in either *CKB2* or *EXO1*. The status of *CKB2* or *EXO1* is indicated across the top of each set of patches, and the bait mutations tested are indicated to the left of each set of patches.

Our strategy for identifying new GIS genes was to generate mutant strains using an adaptation of the Synthetic Genetic Array (SGA) method^34^ and test them for increased genome instability. We crossed a collection of candidate mutant strains (described below) against strains containing one of three GCR assays (GCR query strains; Fig. 1a) and against strains containing a GCR assay and one of 43 mutations (GCR+mutation query strains). The 43 GCR+mutation query strains were included in the crosses because some genes are cooperating Genome Instability Suppressing (cGIS) genes in which mutations only affect genome stability when combined with other mutations^10^. The 43 mutations affected known GIS genes and genes that clustered with known GIS genes^29^ and were selected to maximize the number of gene clusters surveyed (Fig. 1b,c).

The GCR assays select haploid cells resistant to both canavanine (Can) and 5-fluoroorotic acid (5FOA) due to loss of the *CAN1* and *URA3* genes on the left arm of chromosome V (ref. 6). These GCRs have a breakpoint between the *CAN1* and *URA3* genes and the most telomeric essential gene on the left arm of chromosome V (*PCM1*); genomic features in this breakpoint region influence the types of GCRs that are formed^7^,^8^. The short repeated sequence GCR (sGCR) assay contains single-copy sequences in the breakpoint region and ∼100 bp of *YCLWdelta5* sequence in the *can1::P*_*LEU2*_*-NAT* locus that has homology to the long-terminal repeats from Ty1 and Ty2 retrotransposons (Fig. 1a). The segmental duplication GCR (dGCR) assay contains the ∼4-kb *DSF1-HXT13* segmental duplication with divergent homology to regions of chromosomes IV, X and XIV (ref. 8) in addition to the *YCLWdelta5* fragment (Fig. 1a). The Ty912-containing GCR (tyGCR) assay detects GCRs mediated by homologous recombination (HR) with the other Ty-related sequences in the genome (Fig. 1a)^7^. Single and double-mutant haploid strains generated by the SGA procedure were tested for increased accumulation of GCRs by determining the number of Can^R^ 5FOA^R^ papillae observed after growing patches from independent spore clones and replica plating the patches onto GCR selection media (Fig. 1d,e). A numerical score (0–5) was assigned to each patch by counting the total number of papillae per patch, and a GCR strain score was calculated by averaging the scores for all of the patches analysed for each mutant (Fig. 1d). The GCR strain scores are not the direct equivalent of GCR rates; doubling of the strain score corresponds to an increase in GCR rate of an average of fivefold.

As determining GCR strain scores is labour intensive, we implemented a two-stage genetic screening strategy to focus on a subset of non-essential *S. cerevisiae* genes that were enriched in GIS and cGIS genes. The first stage was our previous genome-wide *in silico* screen that identified 1,041 candidate GIS genes^29^. A preliminary investigation of ∼10% of these 1,041 genes identified 34 new GIS genes and 1 new cGIS gene, revealing that this group of genes was enriched for GIS genes but that not all of the 1,041 genes were GIS genes^29^. In the second stage of the screen, which is described in the present study, we eliminated all essential genes from the candidate list of GIS genes and added in all additional non-essential genes known to function in the pathways identified by the 1,041 genes (see Methods; Supplementary Data 1) resulting in 1,055 genes/mutations. Finally, we added two additional *mrc1* and *rad53* alleles and a *leu2Δ* control deletion for a total of 1,058 strains.

The first-generation set of 1,058 mutant strains was crossed to the wild-type dGCR, sGCR and tyGCR query strains and dGCR+query mutation strains containing *dia2Δ*, *exo1Δ*, *rrm3Δ* and *rtt107Δ* mutations, and the resulting progeny were evaluated for increased GCR rates using patch tests (see Methods). On the basis of these results, we generated a second-generation set of 639 mutant strains (see Methods and Supplementary Data 1). This collection of mutants contained all of the mutations that either increased the GCR rate in at least 1 GCR assay or interacted with at least 1 of the *dia2Δ*, *exo1Δ*, *rrm3Δ* and *rtt107Δ* mutations in the dGCR assay. The *dia2Δ*, *exo1Δ*, *rrm3Δ* and *rtt107Δ* mutations were selected for evaluating candidate enhancing mutations because together they interacted with the largest number of bait mutations in a subset of the first-generation set of mutant strains (see Methods). The 419 bait mutations excluded were identified in the *in silico* screen on the basis of causing increased sensitivity to DNA-damaging agents^29^, which can reflect processes unrelated to DNA repair like small molecule export and detoxification. Consistent with this, the 419 excluded genes showed little if any genetic similarity to *bona fide* GIS genes^29^ and were enriched for roles in the endosome, Golgi complex, the ESCRT complex, the retromer complex and general metabolism but not in DNA or chromosome metabolism. Crossing of the second-generation set of mutants to the remaining 39 dGCR+query mutation strains was then continued, and the resulting double-mutant progeny were evaluated for increased GCR rates using patch tests (see Methods).

### Identification of GIS genes

**Figure 2:**
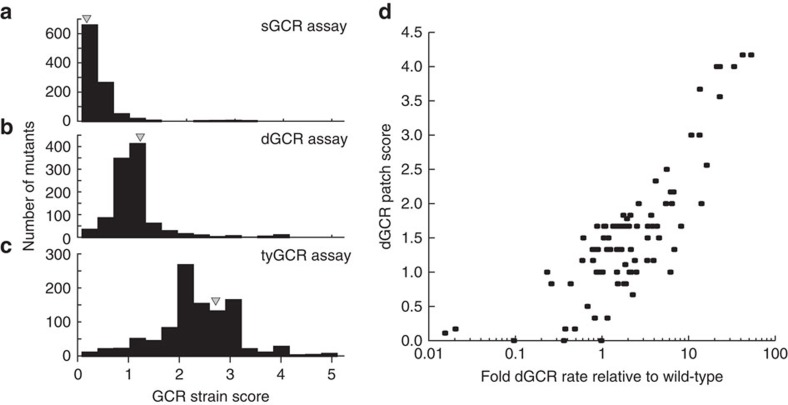
Assaying single-mutant strains using GCR strain scores. (**a**–**c**). Histograms of the distribution of GCR strain scores for single-mutant strains from the sGCR (**a**), dGCR (**b**) and tyGCR (**c**) assays reveal that the average GCR strain score increases with the GCR rate for each GCR assay and that the score of the *leu2Δ* control strain (grey triangle) generally lies at the peak of each histogram, suggesting that many of the mutations tested do not substantially affect the GCR strain score as single mutations. (**d**). The fold increase in the GCR rate is correlated with the GCR strain score for systematically generated strains containing the dGCR assay.

Crossing the wild-type dGCR, sGCR, and tyGCR query strains to the first-generation mutant set generated 1,002, 995, and 1,009 single-mutant strains, respectively (Supplementary Data 1). The GCR strain scores for the *leu2Δ* control strains were 0.1, 0.94, and 2.67 for the sGCR, dGCR, and tyGCR assays, respectively, and were consistent with quantitative GCR rate measurements (Supplementary Data 1). The distribution of strain scores for all of the mutations tested in each GCR assay peaked around the strain scores for the *leu2Δ* control strains, suggesting that most of the individual mutations tested did not strongly affect genome instability (Fig. 2a–c).

To determine a cutoff score for identifying mutations causing increased GCR rates, we determined GCR rates for 101 single-mutant dGCR strains and all 43 *leu2Δ queryΔ* double-mutant dGCR strains from crosses with the dGCR+query mutation strains (Supplementary Tables 1 and 2). We found a robust correlation between the GCR strain scores and GCR rates (Fig. 2d), despite a small but consistent increase in dGCR rates that was observed in strains from the systematic crosses, which was potentially due to GCRs mediated by the *YCLWdelta5* fragment at the *can1::P*_*LEU2*_*-NAT* locus (Supplementary Tables 2 and 3; Supplementary Fig. 1). Using the GCR strain scores and rates for the 144 systematically generated dGCR assay-containing strains, we determined that a cutoff score of 1.4 (0.4 above the wild-type score) balanced the false-positive and false-negative errors in identifying mutations in GIS genes (Supplementary Fig. 2; Methods).

**Figure 3:**
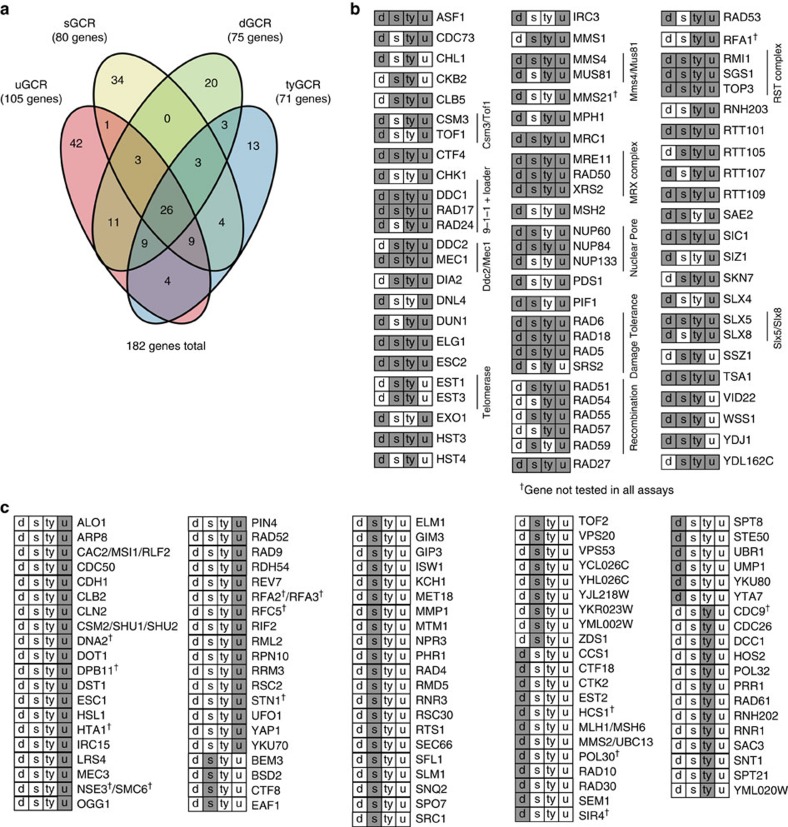
Summary of the increased GCR rates of single-mutant strains identified using patch tests. (**a**). Venn diagram indicating the number of genes that suppress GCRs in each of the GCR assays used. (**b**). Genes implicated in suppressing GCRs in more than one GCR assay. The boxes indicate the assays (d=dGCR, s=sGCR, ty=tyGCR, u=uGCR) in which the listed gene suppresses (grey) or does not suppress (white) GCRs. Note that uGCR assays are GCR assays lacking repetitive sequences in the GCR breakpoint region that have been utilized in previous studies^6^,^8^. Many genes unique to the uGCR assay are primarily genes in which mutations cause small but significant increases in GCR rates, which were identified using fluctuation assays but are difficult to identify by the semi-quantitative patch score method used here. (**c**). Genes implicated in suppressing GCRs in only one GCR assay, annotated as in **b**.

We generated a comprehensive list of GIS genes by combining the GIS genes identified here with those previously known. Initially, we selected all single mutations that caused GCR strain scores that were 0.4 or more above the wild-type score in any GCR assay (Supplementary Data 1; Supplementary Fig. 3). We then removed mutations that caused less than a threefold increase in GCR rate and included mutations that caused at least a threefold increase in rate, regardless of GCR strain scores (Supplementary Tables 2, 4 and 5; Supplementary Fig. 4). Finally, we included mutations previously shown to increase the GCR rate by threefold or more, including mutations in essential genes not studied here and mutations in genes identified in studies using GCR assays lacking repetitive sequences in the GCR breakpoint region (single-copy or unique sequence GCR assays; designated here as uGCR assays^6^,^8^ and previously summarized^29^; Supplementary Data 1). We observed 75 genes that suppressed GCRs in the dGCR assay, 71 genes in the tyGCR assay, 80 genes in the sGCR assay and 105 genes in the uGCR assays. The higher number of GCR suppressing genes identified in the uGCR assays is primarily the result of candidate gene studies that included alleles of essential genes not tested here and mutations that cause small but significant increases in quantitative GCR assays, which were too small to reliably detect by the semi-quantitative scoring method used here. Altogether, we identified 182 *S. cerevisiae* GIS genes, 50 of which suppress genome instability in at least 3 of the 4 GCR assays (Fig. 3; Supplementary Data 1).

This analysis identified 64 previously unrecognized GIS genes, reidentified 62 known GIS genes including 20 identified in our previous test validation^29^, and failed to reidentify 56 previously recognized GIS genes. Of the 56 genes that were not reidentified in this screen, 13 were not discoverable, as these genes were either essential for viability or mating, and 43 caused only a small increase in GCR rate that could not be easily identifiable by patch scores. Fourteen of these 43 genes were found in our previous test validation^29^. Forty two of these 43 were subsequently found as interactors in our cGIS screen (see below), which would be expected to identify weak alleles as interacting mutations. In total, this study and our previous test validation of the list of candidate GIS genes^29^ identified 98 new GIS genes that were not known when we constructed the candidate list^29^. Examples of previously unrecognized GIS genes included *VID22* and *YDJ1*. *VID22* encodes a partner of Tbf1 involved in transcriptional regulation^35^,^36^ and DSB repair^37^. *YDJ1* encodes the major cytosolic Hsp40/DnaJ co-chaperone that acts in protein maturation and stabilization^38^. The imperfect overlap of mutations causing increased GCR strain scores in the different assays suggests that some mutations have different effects on GCRs in different genomic contexts^8^, which was verified by GCR rate measurements (Supplementary Table 1).

To determine the efficiency of our preselection of candidate GIS genes^29^, we crossed the dGCR assay strain to five randomly selected 96-well plates of mutant strains from the *S. cerevisiae* deletion collection and determined GCR strain scores for the progeny (Supplementary Data 1). Only 1 of the 463 single mutants scored, *ydl118wΔ*, which was not previously identified, caused an increased GCR strain score. This deletion was tested in the initial cross but did not cause an increased GCR strain score, likely because it only causes a small increase in GCR rate. Extrapolating to the entire deletion collection, we estimate that our method potentially missed approximately eight GIS genes and that we identified 96% of the GIS genes. However, *ydl118wΔ* was identified in the cGIS gene screen described below; this suggests that at least some of the approximately eight GIS genes that were predicted to not be identified in the single-mutant screen were likely identified in the cGIS gene screen.

### Identification of cGIS genes

**Figure 4:**
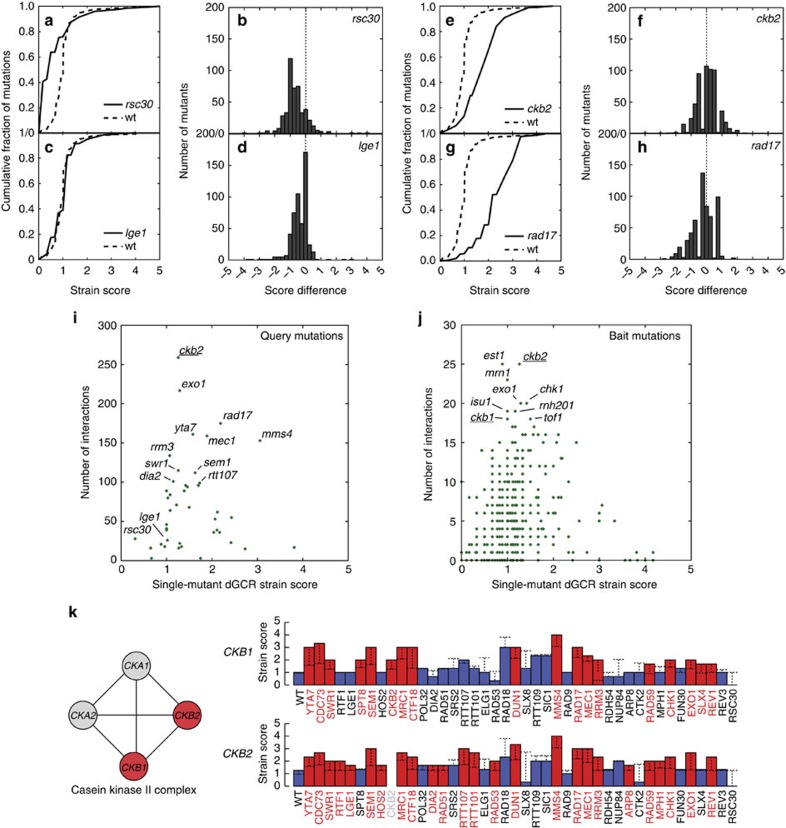
Identification of genetic interactions involved in suppressing genome instability. (**a**,**c**,**e**,**g**). Plots of the cumulative fraction of mutations below specific GCR strain scores for strains containing bait mutations and in addition one of the *rsc30Δ*, *lge1Δ*, *ckb2Δ* or *rad17Δ* query mutations (solid line) compared with the distribution from the crosses of the bait mutations to the wild-type strain (dashed line). (**b**,**d**,**f**,**h**). Histograms of the number of mutations in combination with *rsc30Δ*, *lge1Δ*, *ckb2Δ* or *rad17Δ* as a function of the GCR strain score difference, which is the GCR strain score of the double-mutant strain (*aΔ bΔ*) minus the GCR strain score of the higher of the two single-mutant strains (*aΔ* or *bΔ*). (**i**). Plot of the number of GCR-based interactions as a function of the single-mutant GCR strain score for the 43 mutant query strains. Query mutations with large numbers of interactions or those displayed in **a**–**h** are indicated. (**j**). Plot of the number of GCR-based interactions as a function of the single-mutant GCR strain score for bait mutations. Bait mutations with large numbers of interactions are indicated. (**k**). Analysis of physical interaction data for the casein kinase II complex is shown (left) with reported physical interactions in BioGrid (lines) between complex components (circles). Components with known GCR interactions are in red; untested components (*CKA2*) or those tested with only four query mutations (*CKA1*) are in grey. Display of the genetic interactions between the *ckb1Δ* and *ckb2Δ* bait mutations and the 43 query mutations (right). Bar heights indicate the strain score for the double mutant, and bar colours correspond to the presence (red) or absence (blue) of an increased level of genome instability in the double mutant as observed in patch tests relative to the respective single mutant with the highest level of increased genome instability; the horizontal dashed line corresponds to the GCR strain score of the higher of the two single mutations. Missing bars and query names in grey correspond to double-mutant strains that were not generated in the crosses performed.

We recovered and tested 25,974 double mutants from the crosses of the 43 dGCR+query mutation strains with the first-generation (*dia2Δ*, *exo1Δ*, *rrm3Δ* and *rtt107Δ*) and second-generation (the remaining 39 dGCR+query mutation strains) bait strain sets (Methods; Supplementary Data 1). As the 43 query mutations were also present as bait mutations, we obtained 801 pairs of double-mutant strains (out of a possible 903) generated as both query × bait or bait × query combinations. The individual pairs of these double mutants had consistent GCR strain scores (Supplementary Fig. 5). The scores of the double-mutant strains were distributed about the score of the query mutants as for the single-mutant strains, including mutations causing reduced scores (for example, *rsc30Δ*), scores essentially identical to wild-type (for example, *lge1Δ*) or increased scores (for example, *ckb2Δ* and *rad17Δ*) (Fig. 4a–h; Supplementary Figs 6–11).

Double-mutant strains (*n*=3,149 (∼13%)) that had GCR strain scores that were at least 0.4 (the single-mutant strain differential score) greater than the higher of the two single-mutant strain scores were suggestive of a genetic interaction causing a greater than additive increase in GCR rate (Supplementary Data 1). GCR rate determination of 66 selected double mutants predicted to show a genetic interaction revealed that 71% of these double-mutation combinations resulted in a synergistic increase in GCR rate compared with that of the respective single mutants. Thus, increased double-mutant GCR strain scores were a good indicator for synergistic interactions (Supplementary Table 6). Raising the strain score differential above 0.4 did not substantially improve the identification of synergistic interactions; this suggests that the selection of false-positive double mutants reflects some biological property of the double mutants (for example, selection of suppressor mutations) affecting the patch scores or rates rather than an inappropriate cutoff score. Despite this, double-mutant GCR strain scores reidentified many previously known genetic interactions^10^,^19^, such as the redundancy between the *REV1*–*REV3*–*REV7*- and *MMS2*–*UBC13*-dependent branches of post-replication repair, the dependence on *SRS2* of the increases in GCR rates caused by *rad18Δ* and *rad5Δ* single mutations, and the redundancy of *MEC1*- and *TEL1*-mediated suppression of the formation of GCRs as well as many new interacting mutations (Supplementary Fig. 12).

The query mutations interacting with the largest number of mutations were *ckb2Δ*, *exo1Δ*, *rad17Δ*, *yta7Δ*, *mec1Δ*, *mms4Δ* and *rrm3Δ* (Fig. 4i), and the bait mutations interacting with the largest number of query mutations were *est1Δ*, *ckb2Δ*, *mrn1Δ*, *exo1Δ*, *chk1Δ*, *isu1Δ*, *rnh201Δ*, *ckb1Δ* and *tof1Δ* (Fig. 4j). Two mutations illustrating the complexity of these interactions were *ckb2Δ* and *exo1Δ*, which both interacted with checkpoint defects and also interacted with each other (Supplementary Data 1; Supplementary Table 7), indicating that casein kinase II and Exo1 function in different GCR suppressing pathways, both of which interact with checkpoint pathways. Mutations causing very high (>3) GCR strain scores as single mutations tended to have few interactions, possibility due to difficulties in scoring strains that come close to saturating the assay. In total, 595 mutations interacted with at least one query mutation; 438 of the affected genes were distinct from the 182 GIS genes and hence were cGIS genes (Supplementary Data 1). In total, mutations in 620 genes (182 GIS genes and 438 cGIS genes; 13% of the 4,848 non-essential *S. cerevisiae* ORFs) were identified as causing or enhancing genome instability.

To identify the most robust interactions, we searched for interactions between a query mutation and mutations in multiple genes encoding components of an annotated complex or pathway, which we termed ‘modules' (Fig. 4k; Methods). We found shared interaction for 77 modules (Table 1). Mutations affecting an additional 91 modules had interactions that were not shared (Supplementary Data 2); although this included 64 complexes where only a single gene was tested. Mutations affecting only two modules, Mre11-Rad50-Xrs2 and Sgs1-Top3-Rmi1, caused significant increases in GCR rates but lacked interactions with other mutations; the lack of interacting mutations in these cases was likely due to the fact that single mutations in the genes encoding these complexes cause high GCR strain scores that saturate the assay (∼4.0).

### Inactivation of GIS genes in human cancers

To determine whether defects in GIS genes might occur in cancer, the ovarian cancer, colorectal cancer and acute myeloid leukaemia (AML) TCGA data were analysed^30^,^31^,^32^. The genes analysed were the human homologues of the 182 *S. cerevisiae* GIS genes plus 13 additional genes that act in pathways and protein complexes defined by the GIS genes (hGIS1, 214 genes; Supplementary Data 3) and an expanded list (hGIS2, 279 genes; Supplementary Data 3) that included human DNA repair genes that function in pathways identified in *S. cerevisiae* but lack an *S. cerevisiae* homolog (for example, *BRCA1* and *BRCA2*) or have an *S. cerevisiae* homolog that was not initially identified because of a borderline score (for example, *NHEJ1* and *H2AFX*).

**Figure 5:**
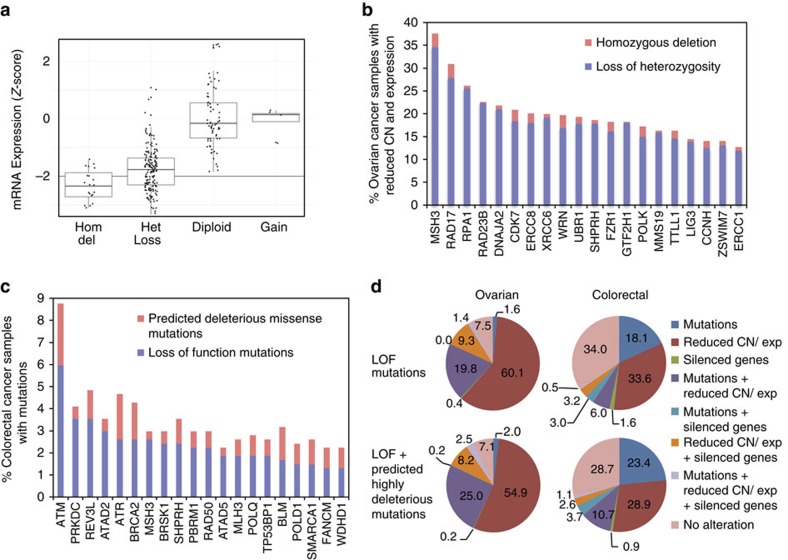
Analysis of the ovarian and colorectal cancer TCGA data for alterations in GIS genes. This figure summarizes the data analysis presented in Supplementary Table 8 and Supplementary Data 4–8. (**a**). Box plot of the RNA Seq data for the copy number (GISTIC −2, Homozygous Deletion; GISTIC −1, Heterozygous Loss; GISTIC 0, Diploid; GISTIC 1, Gain) versus the *Z*-score for mRNA expression of *RAD17* in ovarian cancer. (**b**). Histogram of the frequency of reduced copy number with reduced mRNA expression for the top 20 most-altered GIS genes in ovarian cancer. (**c**). Histogram of the frequency of mutations in the top 20 most-altered GIS genes in colorectal cancer. Data for *MSH2*, *MSH6* and *MLH1* were excluded as defects in these genes predominantly cause increased rates of accumulation of point mutations. Predicted deleterious missense mutations are those that scored as deleterious in 5 or 6 out of 6 functional prediction tests. (**d**). Pie charts showing the % of ovarian (left) and colorectal (right) cancer samples with different combinations of mutations, reduced copy number with reduced expression and silencing among all samples for which any type of genomics data were available. Analysis of LOF mutations alone (Top) and LOF+predicted deleterious missense mutations (Bottom) are presented separately. Note that 19% of the ovarian and 25% of the colorectal cancer cases were not analysed for all types of potential alterations, and consequently the values presented are an underestimate.

To identify potential cancer genes, we used a scoring system (S-score)^39^ that integrates genome-wide data (copy-number variation, expression, methylation and mutations) from a set of tumour samples. In the first analysis, human GIS genes were analysed for signatures consistent with tumour suppressors (S-scores≤−2) or proto-oncogenes (S-scores⩾2; Supplementary Data 3). Genes from hGIS1 and hGIS2 with S-scores≤−2 were enriched in ovarian cancer cases (hGIS1, 26 genes, *P*=0.0008; hGIS2, 41 genes, *P*<0.0001). In contrast, there was no enrichment in human GIS genes with S-scores⩾2 in the ovarian cancer cases (hGIS1, 43 genes, *P*=0.31; hGIS2, 54 genes, *P*=0.40), and these genes were not studied further. The 41 genes from hGIS2 with S-scores≤−2 and 4 additional genes with S-scores between −2 and −1.95 in ovarian cancer were analysed for reduced copy number (GISTIC scores of −1 or −2) associated with reduced expression (*Z*-scores<−2). Reduced copy number associated with reduced expression of 1 to 19 of these 45 genes was observed in 97% of 527 ovarian cancer cases (Supplementary Table 8; Supplementary Data 4). A box plot of the data for one such gene, *RAD17*, and the frequency of occurrence for the top 20 such genes in ovarian cancer are shown in Fig. 5a,b. There were also three genes that appeared to be silenced in 12% of 537 ovarian cancer cases (Supplementary Table 8; Supplementary Data 4). Genes with S-scores≤−2 were enriched (hGIS1, 18 genes, *P*=0.0001; hGIS2, 18 genes. *P*=0.0015) in colorectal cancer cases; in contrast, human GIS genes with S-scores⩾2 were not enriched (hGIS1, 12 genes, *P*=0.10; hGIS2, 16 genes, *P*=0.058) and were not studied further. The 18 genes with S-scores ≤−2 and 2 genes with S-scores between −2 and −1.95 in colorectal cancer cases were further analysed. Reduced copy number associated with reduced expression of 1 to 8 of these 20 genes was observed in 54% of 456 colorectal cancer cases, and 4 genes had apparent silencing in 10% of 463 colorectal cancer cases (Supplementary Table 8; Supplementary Data 5). In the case of AML (222 samples), there was no enrichment of human GIS genes with S-scores≤−2 in hGIS1 (*P*=0.067) and a marginal enrichment of human GIS genes with S-scores≤−2 in hGIS2 (*P*=0.045). There was no enrichment for human GIS genes with S-scores⩾2 in both hGIS1 (*P*=0.085) and hGIS2 (*P*=0.194) and no genes with apparent silencing were identified.

In the second analysis, the number of potential loss-of-function (LOF) mutations (nonsense mutations, frameshift insertion/deletions, in-frame insertion/deletions and splice-site mutations) in the hGIS1 and hGIS2 genes was tabulated for 476 ovarian cancer cases and 537 colorectal cancer cases. For ovarian cancer, LOF mutations were not enriched in hGIS1 genes (*P*=0.87) but were enriched in hGIS2 genes (*P*<0.0001); this increase in significance was due to the presence *BRCA1* and *BRCA2* in the hGIS2 gene list, which accounted for 70% of the LOF mutations in hGIS2 genes. Analysis of the enrichment of classes of the LOF mutations for the hGIS2 genes in ovarian cancer revealed that deletions (includes frameshift deletions; *P*<0.0001), insertions (includes frameshift insertions; *P*<0.0001), frameshift deletions (*P*<0.0001), frameshift insertions (*P*<0.0001) and nonsense mutations (*P*=0.0015) were present at significantly increased levels; many but not all of these LOF mutations were in *BRCA1* or *BRCA2* (Supplementary Data 3). Overall, 27% of the 476 ovarian cancer samples had LOF mutations in at least 1 of 44 predicted human GIS genes, with 1–3 genes mutated per sample (Supplementary Table 8; Supplementary Data 6). LOF mutations in both sets of GIS genes were enriched in the colorectal cancer TCGA cases (hGIS1, *P*<0.0001; hGIS2, *P*=0.0012). The frequency of LOF and predicted deleterious missense mutations for the top 20 hGIS2 genes in colorectal cancer is shown in Fig. 5c. Deletions (including frameshift deletions; hGIS1, *P*=0.0004; hGIS2, *P*=0.0038), mononucleotide repeat frameshifts (hGIS1, *P*<0.0001; hGIS2, *P*<0.0001) and splice-site mutations (hGIS1, *P*=0.0003; hGIS2, *P*=0.0002) were present at significantly increased levels in both hGIS1 and hGIS2 genes, and nonsense mutations were present at statistically significant increased levels in hGIS1 and at borderline significant levels in hGIS2 (hGIS1, *P*=0.01; hGIS2, *P*=0.0613) (Supplementary Data 3). A proportion of colorectal cancer has mismatch repair (MMR) defects associated with high rates of accumulating mutations^32^. We therefore repeated the analysis using a sample set in which the MMR-defective cases had been excluded and found that deletions (including frameshift deletions; hGIS1, *P*=0.001; hGIS2, *P*<0.0001), nonsense mutations (hGIS1, *P*=0.001; hGIS2, *P*=0.0046), frameshift deletions (hGIS1, *P*=0.032; hGIS2, *P*=0.029), mononucleotide repeat frameshifts (hGIS1, *P*=0.0016; hGIS2, *P*=0.0042) and splice-site mutations (hGIS1, *P*=0.0004; hGIS2, *P*<0.0001) were present at significantly increased levels in both hGIS1 and hGIS2, and frameshift insertions were present at significantly increased levels in only hGIS2 (*P*=0.022). This indicates that the accumulation of these classes of mutations in the colorectal cancer cases was not due to MMR defects. Overall, 30% of the 537 colorectal cancer samples had LOF mutations in at least 1 of 185 predicted human GIS genes, with 1–36 genes mutated per sample (Supplementary Table 8; Supplementary Data 7). In the case of AML, there was no enrichment of LOF mutations in the GIS genes (hGIS1, *P*=1.00; hGIS2, *P*=0.99), and as a result, individual classes of mutations were not analysed.

All of the gene inactivation data were merged, and the proportion of different classes of gene inactivation was determined (Fig. 5d; Supplementary Data 8). LOF mutations and LOF mutations plus those missense mutations that scored as ‘predicted deleterious' in at least 5 of 6 function prediction tests were considered separately. In ovarian cancer, the gene inactivation signature was dominated by cases with reduced copy number associated with reduced expression. Colorectal cancer showed a different pattern with less overlap between the cases with mutations and the cases with reduced copy number associated with reduced expression. Overall, when only LOF mutations were considered, a minimum of 93% of ovarian cancer cases and 66% of colorectal cancer cases had a signature of inactivation of one or more predicted GIS genes (Fig. 5d), although these figures are an underestimate because not all samples were analysed for all types of alterations. It should be noted that the colorectal cancer cases did include cases with alterations in MMR genes (*MSH2*, *MSH6*, *MSH1* and *PMS2*), including 46 cases with only LOF mutations and 50 cases with LOF+predicted deleterious missense mutations (19 of these cases had silencing of *MLH1*, 1 of which also had LOH of *MLH1*), all but 3 of which had alterations in other GIS genes. In the ovarian cancer cases, there were 23 cases of reduced copy number and reduced expression of *MLH1*, 3 cases with a LOF mutation in an MMR gene and 2 cases with a predicted deleterious missense mutation in an MMR gene; all of these cases had alterations affecting other GIS genes. This indicates that potential MMR defects account for only a small fraction of the alterations affecting GIS genes; it should be noted that MMR defects cause increased GCR rates in addition to increased mutation rates^8^.

The 103 human homologues of the 98 newly identified *S. cerevisiae* GIS genes were analysed separately (Supplementary Data 9). When only LOF mutations, reduced copy number with reduced expression and silencing were considered 64% of ovarian cancer cases had defects affecting at least 1 of 24 of the 103 human genes, and 47% of colorectal cancer cases had defects affecting at least 1 of 67 of the 103 genes. When predicted highly deleterious missense mutations were included, 65% of ovarian cancer cases had defects affecting at least 1 of 37 of the 103 genes, and 51% of colorectal cancer cases had defects affecting at least 1 of 84 of the 103 genes. This indicates that the newly identified GIS genes likely account for a large number of human GIS genes in which defects can cause increased genome instability in human cancers.

## Discussion

Here we developed methods to screen the *S. cerevisiae* systematic deletion collection to identify new GIS genes, identify genes that interact to suppress the formation of GCRs and identify candidate human genes for the analysis of cancer genomics data to identify potential GIS gene defects in human cancers. This analysis increased the total number of known GIS genes to 182, including 98 new GIS genes identified here and during our targeted validation of the starting 1,041 candidate genes. We also identified 438 cGIS genes and an extensive catalogue of genetic interactions affecting genome stability. Analysis of ovarian and colorectal cancer TCGA data^31^,^32^ showed that the majority of the cancer cases analysed (a minimum of 93% of ovarian and 66% of colorectal cancer cases) appeared to have defects affecting one or more genes that were homologues of the *S. cerevisiae* GIS genes or act in the pathways identified by the GIS genes. In contrast, AML, a cancer with little genome instability^33^, did not appear to have defects affecting GIS genes. Thus, genetic or epigenetic changes causing increased genome instability are likely common in some types of cancer, but due to the large number of GIS genes, the defect signature for any single gene can be weak.

Almost half of the 182 *S. cerevisiae* GIS genes suppress the formation of GCRs detected in multiple GCR assays. The common pathways identified typically include genes involved in DNA metabolism, including DNA replication and repair, and genes involved in checkpoint signalling in response to DNA damage and replication errors. Some of the genes identified, such as *RAD27* and *TSA1*, likely function by suppressing the formation of DNA damage^27^,^40^. Other genes, such as those encoding the Mre11-Rad50-Xrs2 and Sgs1-Top3-Rmi1 complexes, likely process DNA damage generated by other mechanisms^41^, such as DNA replication errors. A number of genes have less clear roles in suppressing genome instability, such as *VID22*, *YDJ1*, *SSZ1* and *CKB2*. The fact that many GIS genes suppress GCRs detected in multiple assays that probe different genomic contexts indicates that these genes can suppress the formation of many types of GCRs^7^,^8^,^10^,^12^,^15^. A notable exception are *pif1* mutations that cause a defect in suppression of *de novo* telomere additions, which appears insensitive to genomic context^8^,^18^,^42^. In contrast, a number of genes suppress GCRs detected by subsets of GCR assays (Fig. 3). In most cases, the mechanisms underlying this specificity are not yet understood; however, in the case of *MSH2* and *MSH6*, the heteroduplexes formed by non-allelic homologous recombination (HR) during the formation of duplication-mediated GCRs are likely to contain a higher density of mispairs and hence be better recognized by MMR and subjected to heteroduplex rejection^7^,^8^ than heteroduplexes formed in the tyGCR and sGCR assays.

Mutations that enhance the accumulation of GCRs can in principle act in compensatory or parallel pathways or can have more complicated relationships involving genes within pathways^43^. In addition, mutations can result in increased levels of DNA damage that can lead to GCRs when repair mechanisms are defective or are saturated by the increased levels of DNA damage. Many mutations showing genetic interactions, such as *exo1Δ*, cause increased GCR rates as single mutations. Mutations like these could affect the response to normal levels of spontaneous DNA damage as well as DNA damage that is either induced in the absence of other pathways or is normally repaired in part by other pathways. In contrast, a number of enhancer mutations, such as *tel1Δ* cause no increase in GCR rates as single mutations^10^. These mutations may either result in increased DNA damage that is efficiently repaired so long as the relevant repair mechanisms are functional and not overwhelmed by other sources of damage or inactivate a redundant pathway. Defects in the genes encoding complexes can show the same types of interactions, regardless of whether defects in all of the genes encoding a complex behave similarly (such as *RNH201, RNH202* and *RNH203* as well as *MMS2* and *UBC13*) or whether defects in only a subset of the genes encoding a complex have similar properties (for example, *SPT3*, *SPT8* encoding part of SAGA).

The systematic identification of *S. cerevisiae* GIS genes has facilitated a pathway-based analysis of human cancer genomics data. We have focused on ovarian and colorectal cancer, two cancers with genome instability that appear to have different relative frequencies of copy-number changes and mutation driver alterations^30^, as well as AML, a cancer that is associated with little if any genome instability^30^. In the case of the ovarian cancer TCGA data, 23% of the samples with any data had LOF mutations in GIS gene homologues, with 65% of the samples with LOF mutations having LOF mutations in *BRCA1* and *BRCA2* as previously reported^31^; no other individual GIS gene homolog had a LOF mutation in >0.5 to 1% of the samples. In contrast, there was a high frequency of copy-number alterations, including both copy-number reductions and homozygous deletions, associated with reduced expression of GIS gene homologues in ovarian cancer. This included 17% of the samples that had homozygous deletions of 1 to 9 GIS gene homologues per sample, approximating the frequency of samples with *BRCA1* or *BRCA2* LOF mutations. In contrast, the colorectal cancer TCGA data showed a higher proportion of samples and GIS gene homologues with LOF mutations and a lower yet high proportion of samples and GIS gene homologues with copy-number alterations associated with reduced expression. A minimum of 93% of ovarian cancer TCGA cases and 66% of colorectal cancer TCGA cases had alterations (not considering predicted deleterious missense mutations) affecting one or more GIS gene homologues, with only 5 and 8% of the samples, respectively, having alterations in genes expected to cause a strong MMR defect (*MSH2*, *MSH6*, *MLH1* and *PMS2*) and hence a mutator phenotype. Overall, these results suggest that a high prevalence of alterations in GIS genes can explain how genome stability is compromised in these two cancers. Consistent with this view, there was no evidence for significant alteration of GIS genes in AML, a cancer that is not associated with high levels of genome instability^30^. Defects in some of the human genes identified here have been implicated in cancer (for example, *BRCA1*, *BRCA2*, *ATM*, *BLM*, *REV3L* and *PBRM1*), and some of the genes have been associated with the suppression of genome instability (for example, *WRN*, *BLM*, *ATM*, *ATR*, *BRCA1* and *BRCA2*) or with pathways thought to act in the suppression of genome instability (for example, *RAD17*, *RAD50*, *XRCC6* and *TP53BP1*)^44^,^45^. Our functional studies in *S. cerevisiae* provide evidence that many of the human GIS gene homologues likely act in the suppression of genome instability in human cells and provide a restricted, prioritized list of human genes for genetics and functional validation studies.

## Methods

### Plasmid construction

The plasmid pRDK1590, which is a version of pRS315 (ref. 46) in which the *LEU2* open reading frame was replaced by the nourseothricin-resistance (*NAT*) open reading frame, was constructed by gap repair in the *S. cerevisiae* strain BY4741 as follows. BY4741 was transformed with *Afl*II-digested pRS315, and the *NAT* open reading frame amplified from plasmid pFA6a-natNT2 using the primers 5′- CTTTTACATTTCAGCAATATATATATATATTTCAAGGATATACCATTCTAatgggtaccactcttgacga -3′ and 5′- ATTTCATTTATAAAGTTTATGTACAAATATCATAAAAAAAGAGAATCTTTttaggggcagggcatgctca -3′, where uppercase letters correspond to pRS315 sequence and the lowercase letters correspond to *NAT* sequence. The plasmid pRDK1593 was generated by sub-cloning a *P*_*LEU2*_*-NAT*-containing BsrGI to XbaI fragment from pRDK1590 into pRS305 (ref. 46) digested with BsrGI and XbaI and was subsequently used as a template for PCR amplification of *P*_*LEU2*_*-NAT* for generating gene disruptions.

### Query strain construction

The selectable markers used in the MATα query strains in systematic mating in the original SGA protocol^34^ are incompatible with the genetic markers required for GCR assays. Therefore, different selectable markers were introduced into MATα query strains containing GCR assays. The selected markers were as follows. First, because the GCR assay requires *CAN1*, which interferes with use of canavanine in combination with thialysine to kill diploid strains in the SGA protocol^34^, we introduced a deletion of *LYP1* and the cycloheximide-resistant *cyh2-Q38K* mutation^47^ into our strains, allowing the use of thialysine and cycloheximide to kill diploid strains in our SGA protocol. Second, we introduced a copy of *LEU2* driven by the *MFA1* promoter near the *YFR016C* gene to select for MAT**a** haploid progeny. Third, we replaced the native *CAN1* gene with a selectable nourseothricin-resistance gene driven by the *LEU2* promoter in the dGCR and sGCR assay strains. The MAT**a** and MATα strains with *P*_*LEU2*_*-NAT* were nourseothricin-resistant when grown on complete synthetic media (CSM; made with dropout mixes from US Biological). However, the MAT**a** strains were not nourseothricin-resistant on YPD (1% Bacto-yeast extract, 2% Bacto-peptone, 2% dextrose) medium, which is potentially due to increased expression of the Leu2 protein in MAT**a** strains, resulting in downregulation of the *LEU2* promoter; this did not interfere with the selection scheme because the selections were performed in the appropriate CSM-dropout media.

The required strains were constructed in the following steps. First, BY404 (MAT**a** *ade2::hisG his3Δ200 leu2Δ0 trp1Δ63 ura3Δ0*) was crossed with RDKY3686 (MATα *hom3–10 lys2-10A his3Δ200 leu2Δ1 trp1Δ63 ura3–52*) and sporulated to isolate RDKY7595 (MAT**a** *lys2-10A hom3–10 his3Δ200 leu2Δ0 trp1Δ63 ura3Δ0*) and RDKY7594, a MATα version of RDKY7595. *URA3* was amplified from pRS306 with the primers 5′- GGAGTTTATGTTTATATACACCGGT GTAGGCTGTGCGTTGGTGTGAACACgagca gattgtactgagagtgcacc -3′ and 5′- GGCTGTATGACTACAGTTGCATGCG GAGACGGCTTCAACAGCAACAGCAActccttacgcat ctgtgcggtatttc -3′ and inserted 3' to the *YFR016C* gene to generate RDKY7596. The *iYFR016C::URA3* insertion was then replaced with a *P*_*MFA1*_*-LEU2* construct amplified from FYAT258, generously provided by D. Bernard^48^, using the primers 5′- GGA GTT TAT GTT TAT ATA CAC CGG TGT AGG CTG TGC GTT GGT GTG AAC ACg taa caa tag atc cac tag -3′ and 5′- GGC TGT ATG ACT ACA GTT GCA TGC GGA GGC TTC AAC AGC AAC AGC Aaa ttt aag tat tca ctt tcg -3′ to generate RDKY7597 (MAT**a** *lys2-10A hom3–10 his3Δ200 leu2Δ0 trp1Δ63 ura3Δ0 iYFR016C::P*_*MFA1*_*-LEU2*). RDKY7594 was crossed to RDKY7597 and sporulated to generate RDKY7598, a MATα version of RDKY7597. *HXT13* was replaced by *URA3* in RDKY7598 to generate RDKY7599. A wild-type copy of *LYS2* was amplified from BY4741 and used to replace the *lys2-10A* allele in RDKY7599 to generate RDKY6970. The *LYP1* gene in RDKY6970 was then replaced by *TRP1* to generate RDKY6971. A *cyh2* mutation, determined to be *cyh2-Q38K* by sequencing, was selected in RDKY6971 on YPD plates containing 10 μg ml^−1^ cycloheximide (Sigma) to generate RDKY6975 (MATα *hom3–10 his3Δ200 leu2Δ0 trp1Δ63 ura3Δ0 lyp1::TRP1 iYFR016C::P*_*MFA1*_*-LEU2 cyh2-Q38K hxt13::URA3*). RDKY6975 and RDKY7597 were crossed, and the resulting diploid was sporulated to obtain RDKY7625 (MATα *hom3–10 his3Δ200 leu2Δ0 trp1Δ63 ura3Δ0 lyp1::TRP1 iYFR016C::P*_*MFA1*_*-LEU2 cyh2-Q38K*). The *P*_*LEU2*_*-NAT* gene was amplified from pRDK1593 and integrated into the *CAN1* locus in RDKY7625 to generate RDKY7629. The *CAN1/URA3* cassette with flanking targeting sequences was amplified from pRDK1378 and pRDKY1379 and integrated into RDKY7629 to generate the dGCR query strain RDKY7635 (MATα *hom3–10 ura3Δ0 leu2Δ0 trp1Δ63 his3Δ200 lyp1::TRP1 cyh2-Q38K iYFR016C::P*_*MFA1*_*-LEU2 can1::P*_*LEU2*_*-NAT yel072w::CAN1/URA3*) and the sGCR query strain RDKY7964 (MATα *hom3–10 ura3Δ0 leu2Δ0 trp1Δ63 his3Δ200 lyp1::TRP1 cyh2-Q38K iYFR016C::P*_*MFA1*_*-LEU2 can1::P*_*LEU2*_*-NAT yel068c::CAN1/URA3*), respectively (Supplementary Table 9). The tyGCR assay strain was constructed by crossing RDKY6975 with RDKY6593 (ref. 7) and sporulating the resulting diploid to recover RDKY7046 (MATα *hom3–10 ura3Δ0 leu2Δ0 trp1Δ63 his3Δ200 lyp1::TRP1 cyh2-Q38K iYFR016C::P*_*MFA1*_*-LEU2 iYEL062W::Ty912-hphNT1 hxt13::URA3).* Disruption of the 43 query genes in RDKY7635 with *HIS3* was performed using standard methods (Supplementary Table 9).

### First-generation set of bait strains

The first-generation set of bait strains (Supplementary Data 1) was primarily obtained from strains present in the *S. cerevisiae* deletion collection (Open Biosystems). The mutant strains were chosen based on the 1,041 genes identified in our *in silico* screen for candidate GIS genes^29^ (Supplementary Data 1). Among the 1,041 genes, 46 genes were not included; the majority of these 46 genes were either essential for viability or sporulation or encoded *TLC1*, which is a non-protein-coding gene and therefore not present in the available deletion collection (Supplementary Data 1). An additional 12 genes were not included because the 1,041 genes in the *in silico* screen were finalized after the first-generation bait strain set was selected (Supplementary Data 1). Mutations in some of the 1,041 candidate GIS genes were not present in the deletion collection and were subsequently constructed in BY4741, including *mec1::G418 sml1::hph*, *ddc2::G418 sml1::hph*, *rad53::G418 sml1::hph*, and *mrc1-aq.G418*. In addition, we constructed a control strain by replacing *leu2Δ*0 present in BY4741 with the G418-resistance marker, which allows *leu2::G418*-containing progeny to be selected during systematic mating; these control strains are labelled as *leu2Δ* in the figures. We also added mutations in 60 additional genes associated with pathways implicated by the 1,041 genes identified in the *in silico* screen (Supplementary Data 1). We verified all the deletions by PCR amplification using primers that hybridized within the inserted G418-resistance cassette and primers that hybridized to flanking sequences. Deletions that could not be verified were either replaced by crossing a verified BY4742 deletion strain with BY4741 and sporulating the resulting diploid or by constructing new strains by PCR-mediated gene disruption in BY4741 when a verified BY4742 strain was unavailable (Supplementary Table 10). The final first-generation mutation strain set included 1,058 strains (corresponding to deletions of 1,055 genes of interest with two additional *mrc1* and *rad53* alleles and the *leu2Δ* control deletion; Supplementary Data 1).

### Second-generation set of bait strains

To facilitate double-mutant strain production, we divided the first-generation bait strain collection into two groups, (i) a ‘high-priority' set (502 strains) and (ii) a ‘low-priority' set (555 strains; Supplementary Data 1). The high-priority set contained mutations in GIS genes and genes with patterns of genetic interactions that were most similar to those of known GIS genes^29^ (Supplementary Fig. 13). During the initial construction and analysis of double-mutant strains, we identified four mutations, *dia2Δ*, *exo1Δ*, *rrm3Δ* and *rtt107Δ*, out of 30 mutations tested at the time, which interacted with the largest number of bait mutations in the high-priority set, resulting in increased GCR strain scores. No other set of the final 43 query mutations interacted with >90% of the mutations that the *dia2Δ*, *exo1Δ*, *rrm3Δ* and *rtt107Δ* mutations were found to interact with. We crossed these four mutations to the low-priority set of mutants and scored the resulting double mutants. These four query mutations showed genetic interactions with a much lower proportion of the mutations in the low-priority mutation set compared with the high-priority mutation set (Supplementary Fig. 14). We then identified mutations in the low-priority set that (i) increased the GCR strain scores in at least one of the dGCR, sGCR or tyGCR assay-containing strains (22 mutations), (ii) showed interactions with at least one of the *dia2Δ*, *exo1Δ*, *rrm3Δ* and *rtt107Δ* mutations in the dGCR assay (87 mutations, 9 in common with group (i)), or (iii) could not be evaluated as we did not recover strains when crossing the wild-type query strains or dGCR assay+mutant query strains (39 mutations). We then added strains containing these mutations to the strains containing the high-priority mutations. This resulted in a second-generation bait strain collection containing 639 strains that were then crossed to the remainder of the dGCR+query mutation strains.

### Screen for GCR-suppressing genes and interacting genes

Query strains grown on YPD-agar were crossed to arrayed strains containing bait mutations on YPD-agar in quadruplicate by pinning onto a fresh YPD agar plate using a Singer RoToR robot (Singer Instruments, UK) and grown for 1–2 days at 30 °C. The cells were then subjected to two rounds of pinning onto diploid selection medium (YPD-agar containing 200 μg ml^−1^ geneticin (G418; Gibco) and 100 μg ml^−1^ nourseothricin (clonNAT; Werner BioAgents)) and grown for 1–2 days at 30 °C. The cells were then pinned onto presporulation medium (containing 15 g Difco nutrient broth (Fisher Scientific), 5 g Bacto-yeast extract (Fisher Scientific), 10 g Bacto-agar (Fisher Scientific) and 62.5 ml 40% glucose per 500 ml) and grown for 3 days at 30 °C. Cells from the presporulation medium were then pinned onto sporulation medium (10 g potassium acetate, 0.05 g zinc acetate, 20 g Bacto-agar per liter, containing a final concentration of 50 μg ml^−1^ G418 and 25 μg ml^−1^ nourseothricin) and incubated for 7 days at 30 °C. The resulting spore-containing cells were then subjected to two rounds of pinning onto diploid killing medium (1.7 g yeast nitrogen base without amino acids and without ammonium sulfate (Fisher Scientific), 1 g L-glutamic acid monosodium salt (Sigma), 2 g dropout mix^34^ without uracil, lysine, leucine and, when appropriate, without histidine, 20 g Bacto-agar, 50 ml of 40% glucose per liter, containing a final concentration of 50 μg ml^−1^ thialysine (*S*-(2-aminoethyl)-L-cysteine hydrochloride; Sigma), 10 μg ml^−1^ cycloheximide, 200 μg ml^−1^ G418, and 100 μg ml^−1^ nourseothricin) followed by growth for 5 days at 30 °C for the first pinning and 2 days at 30 °C for the second pinning. Cells were then subjected to two rounds of pinning and growth on haploid selection medium (1.7 g yeast nitrogen base without amino acids and without ammonium sulfate, 1 g L-glutamic acid monosodium salt, 2 g CSM dropout mix without leucine, uracil and, when appropriate, without histidine, 20 g Bacto-agar, 50 ml of 40% glucose per liter, containing a final concentration of 200 μg ml^−1^ G418 and 100 μg/ml nourseothricin) and grown for 2 days at 30 °C. Then the cells were pinned and grown on YPD-agar followed by storage at −85 °C in YPD media containing glycerol.

### GCR patch tests

A minimum of three independent spore clones were isolated from each mutant progeny pool arising from the SGA protocol and then grown as patches on a YPD-agar plate at 30 °C for two days and replica-plated onto CSM -Arg media containing 60 mg l^−1^ canavanine (Sigma) and 1 g l^−1^ 5-fluoroorotic acid (US Biological). The number of papillae growing on the GCR medium was scored using a semi-quantitative scoring system as follows: 0, no papillae; 1, 1–5 papillae (this was on average the number of papillae observed with the *leu2Δ* control strain for the dGCR assay); 2, 6–15 papillae; 3, 16—a countable number of papillae (∼150–200); 4, papillae that were too many or too close together to count; 5, a lawn of papillae covering the entire patch (Fig. 1d). Then the scores for all independent patches analysed for each mutant were averaged to generate a GCR strain score (Supplementary Data 1). Negative scores were assigned to strains that did not grow so that these strains could be ignored during the analysis.

### Determination of GCR rates

The media and protocol for strain propagation and measuring GCR rates were as described previously^49^.

### Determination of an optimal cutoff score

Using 101 paired GCR rates and average GCR patch scores for single mutants in the dGCR assay and 43 strains resulting from the crosses of mutant dGCR query strains with the *leu2Δ* control strain, we determined an optimal cutoff score as described^50^. Briefly, for any given cutoff value, *c*_*i*_, we calculated the sensitivity, which is the fraction of mutations causing increased GCR rates that we include as *SENS*_*i*_=*TP*_*i*_ /(*TP*_*i*_ +*FN*_*i*_), where *TP*_*i*_ is the number of true positives (mutants with a GCR rate at least threefold higher than wild-type with a score ⩾*c*_*i*_) and *FN*_*i*_ is the number of false negatives (mutants with a GCR rate at least threefold higher than wild type with a score<*c*_*i*_). For each cutoff value, we also calculated the specificity, which is the fraction of mutants that do not have increased GCR rates that we reject: *SPEC*_*i*_=*TN*_*i*_/(*TN*_*i*_+*FP*_*i*_), where *TN*_*i*_ is the number of true negatives (mutants with a GCR rate less than threefold higher than wild-type with a score<*c*_*i*_) and *FP*_*i*_ is the number of false positives (mutants with a GCR rate less than threefold higher than wild-type with a score>*c*_*i*_). An optimal cutoff for balancing sensitivity and specificity can be determined by optimizing the cost function *w*_*1*_*SENS*_*i*_ *+w*_*2*_*SPEC*_*i*_ as a function of *c*_*i*_. Here we weighted sensitivity slightly higher than specificity (*w*_*1*_*=*2, *w*_*2*_*=*1) with the rationale that false negatives were more problematic because false positives could be identified by subsequent quantitative rate testing. We found that the optimal cutoff *c*_*i*_ was 1.38 for the set of 101 rate/score pairs solely from the wild-type dGCR cross and for the set of 144 rate/score pairs from the wild-type dGCR cross and the *leu2* double mutants from the mutant dGCR crosses (Supplementary Fig. 2). With equal weights, the optimal cutoff was slightly higher, ∼1.69. As expected, analysis of Receiver-Operator Characteristic (ROC) curves^51^ showed that mutations causing higher GCR rates were clearly better detected by these patch-based GCR strain scores than mutations that only weakly increased the GCR rates (Supplementary Fig. 2). We also used the Kolmogorov–Smirnov test as extended for discrete null distributions as implemented in R^52^ to calculate *P*-values for differences between the distribution of patches from the *leu2Δ* control strain and each single mutant. Unlike calculations based on the average GCR patch score, this test included the number and distribution of all observed patches. We found that the list of mutant strains with significantly different patch scores that were higher than the *leu2Δ* control strain (*P*<0.01) was essentially the same as the list of strains identified by minimizing the false-positive and false-negative errors as described above.

### Analysis of *S. cerevisiae* modules

Protein complex and pathway (module) definitions were extracted from a variety of studies^53^,^54^,^55^,^56^,^57^,^58^,^59^,^60^ as well as manually curated complexes such as CYC2008v2 and YHTP2008 (ref. 61), the *Saccharomyces* Genome Database GO complex and pathway definitions^62^, *S. cerevisiae* KEGG pathways^63^, and *S. cerevisiae* MetaCyc pathways^64^. Modules containing genes that showed increased GCR scores alone or enhanced the GCR scores of query mutations were identified. Hits were manually curated to identify well-supported modules, and these modules were divided into two groups. The first group contained modules with more than one gene that when mutated shared at least one query mutation that caused increased GCR scores. The second group contained modules for which only a single gene caused increased GCR interactions when mutated or for which multiple genes when mutated caused increased GCR interactions but lack shared interacting partners.

### Analysis of cancer genomics data

TCGA data^31^,^32^, including expression *z*-scores, methylation and GISTIC CNV (copy number variation) data were obtained from the cBIO portal (http://www.cbioportal.org) through the CGDS-R package. Somatic mutation data were obtained from a local compilation^39^ that includes data from TCGA and COSMIC as well as a compilation of data from the literature. As previously described^39^, all mutations for a given tumour were used in the S-score calculation. For all other analyses, only TCGA mutation data were used. As defined by TCGA, putative copy-number calls on samples were determined using the GISTIC algorithm^65^. Boxplots were generated using ggplot2, a graphics tool for the R statistical package (http://ggplot2.org). For expression data, the *Z*-score metrics adopted by TCGA were used. The data for all tumour samples were categorized in Excel Spreadsheets using the cut-offs for copy number, expression, methylation and mutation predictions indicated in the contents of each Supplementary Data spreadsheet.

### Computational prediction of the functional impact of missense mutations

To identify putative deleterious missense mutations in our gene set, we used five different computational algorithms resulting in six different tests per mutation: SIFT^66^, PolyPhen-2 (ref. 67), MutationTester^68^, Fathmm^69^ and LTR^70^. Two versions of PolyPhen-2 were used, each one trained by a different dataset (HDIV and HVAR). Each missense mutation was assigned a score called the ‘Ndamage score' that was the number of prediction tests in which the mutation scored as deleterious. To be considered ‘predicted deleterious', a given missense mutation had to have an Ndamage score of 5 or 6.

### Simulations to determine statistical significance in cancer genomics analyses

Two types of simulations were used. First, a gene-set enrichment analysis was performed to evaluate whether the set of GIS genes were enriched with genes with extreme S-scores (≤−2 or ⩾2). Ten thousand random sets of the same size (number of genes) were selected from the pool of all human genes, and for each set the number of genes with extreme S-scores was defined. A *P*-value for the enrichment analysis was determined by ranking the real set in the random set distribution. Second, we evaluated whether a given set of genes was enriched for different types of mutations (or combinations of different types). To avoid any bias due to different gene lengths, we normalized the analysis for the total length of the corresponding gene set (in amino acids of the longest coding region for each gene). The total number of amino acids for the real set was randomly selected from the total pool of human genes (10,000 random sets). The number of mutations in the real set was then compared with all random sets, and a *P* value for the enrichment analysis was determined by ranking the real set within the distribution of the random sets.

## Additional information

**How to cite this article:** Putnam, C. D. *et al*. A genetic network that suppresses genome rearrangements in *Saccharomyces cerevisiae* and contains defects in cancers. *Nat. Commun.* 7:11256 doi: 10.1038/ncomms11256 (2016).

## Supplementary Material

Supplementary Information Supplementary Figures 1-14, Supplementary Tables 1-10 and Supplementary References

Supplementary Data 1 GCR scores and bait mutations.

Supplementary Data 2 Modules implicated in the dGCR enhancer screen.

Supplementary Data 3 List of S. cerevisiae and human GIS genes with S-scores and number of mutations (by type) found in each gene.

Supplementary Data 4 Analysis of expression, copy number and methylation for human GIS genes in ovarian cancer.

Supplementary Data 5 Analysis of expression, copy number and methylation for human GIS genes in colorectal cancer.

Supplementary Data 6 Analysis of human GIS gene mutations in ovarian cancer.

Supplementary Data 7 Analysis of human GIS gene mutations in colorectal cancer.

Supplementary Data 8 Combined analysis of mutations, expression, copy number and methylation for human GIS genes in colorectal and ovarian cancer.

Supplementary Data 9 Combined analysis of mutations, expression, copy number, and methylation for the human homologs of the 98 new S. cerevisiae GIS genes in colorectal and ovarian cancer.

## Figures and Tables

**Table 1:** Modules with shared interactions in the dGCR enhancer screen.

**Process**	Module*	
DNA repair	Core mitotic homologous recombination	Mms2–Ubc13 complex
	Base-excision repair	Mms4–Mus81 complex
	Cul8–RING ubiquitin ligase complex	Msh2–Msh6 complex
	DNA ligase IV complex	Nucleotide-excision repair factor 1 (NEF1) complex
	DNA polymerase zeta—Rev1 complex	Nucleotide-excision repair factor 4 complex
	Ku complex	Rad1–Rad10–Saw1 complex
	Mlh1–Mlh2 complex	Ribonuclease H2 complex
	Mlh1–Mlh3 complex	Shu complex
	Mlh1–Pms1 complex	Slx1–Slx4 complex
		
DNA replication	DNA polymerase epsilon complex	
	Ribonucleoside-diphosphate reductase complex	
	Telomerase	
		
Chromosome cohesion and	Ctf18 RFC-like complex	
segregation	Ctf19 complex (includes COMA complex)	
	Dynactin complex	
	Monopolin	
	Msh4–Msh5 complex	
	Prefoldin complex	
		
Cell cycle checkpoints	Anaphase-promoting complex (APC/C)	
	Cdc28 cyclin-dependent kinase complexes	
	Mec1–Ddc2 complex	
	Protein phosphatase (PP4) complex	
	Rad17–Ddc1–Mec3 complex+Rad24-Rfc2–5 clamploader	
	Spindle checkpoint	
	Tof1–Csm3 complex	
		
Chromatin/transcription/	Carboxy-terminal domain protein kinase complex	
mRNA processing	CCR4-NOT core complex	
	Cdc73/Paf1 complex	
	Chromatin assembly complex	
	Chz1–Htz1–Htb1 complex	
	COMPASS complex	
	Cytoplasmic mRNA processing body	
	Cytoplasmic Sm-like complex	
	Elongin–Cullin–Socs (ECS) ligase complex	
	HIR complex	
	Ino80 complex	
	ISW1a chromatin remodelling complex	
	Mediator complex	
	NuA3 histone acetyltransferase complex	
	NuA4 histone acetyltransferase complex	
	Rpd3L complex	
	Rpd3S complex	
	RNA polymerase I complex	
	RSC complex	
	SAGA complex	
	Set3C complex	
	SLIK (SAGA-like) complex	
	Spt3–Spt8 SAGA subunit of SAGA complex	
	Swr1 complex	
	U6 snRNP	
		
Nuclear pore	Nuclear pore nuclear basket	
	Nuclear pore outer ring	
		
Proteasome/protein	Doa10 ubiquitin ligase complex	
degradation	Hrd1p ubiquitin ligase ERAD-L complex	
	proteasome 19/22S regulator	
	proteasome 20S complex+Ump1 chaperone,	
	Rad6–Ubr1 complex	
	Ula–Uba3 complex	
		
Other	AP-3 adaptor complex	
	Casein kinase II complex	
	Chs5p/Arf-1 binding proteins (ChAPs)	
	ESCRT III complex	
	Golgi transport complex	
	HMC complex	
	Kel1–Kel2 complex	
	NatA complex	
	Sod1–Ccs1 complex	
	Ssk1–Ssk2 complex	

dGCR, duplication-mediated gross chromosomal rearrangement assay; mRNA, messenger RNA

^*^See Supplementary Data 2 for genes in each module.
