# Supplementary material for: A genetic network that suppresses genome rearrangements in Saccharomyces cerevisiae and contains defects in cancers
Source: Nat Commun. 2016 Apr 13;7:11256. doi: 10.1038/ncomms11256 (PMC4833866; doi:10.1038/ncomms11256)
Supplement: Supplementary Information — Supplementary Figures 1-14, Supplementary Tables 1-10 and Supplementary References [file ncomms11256-s1.pdf]

Supplementary Information

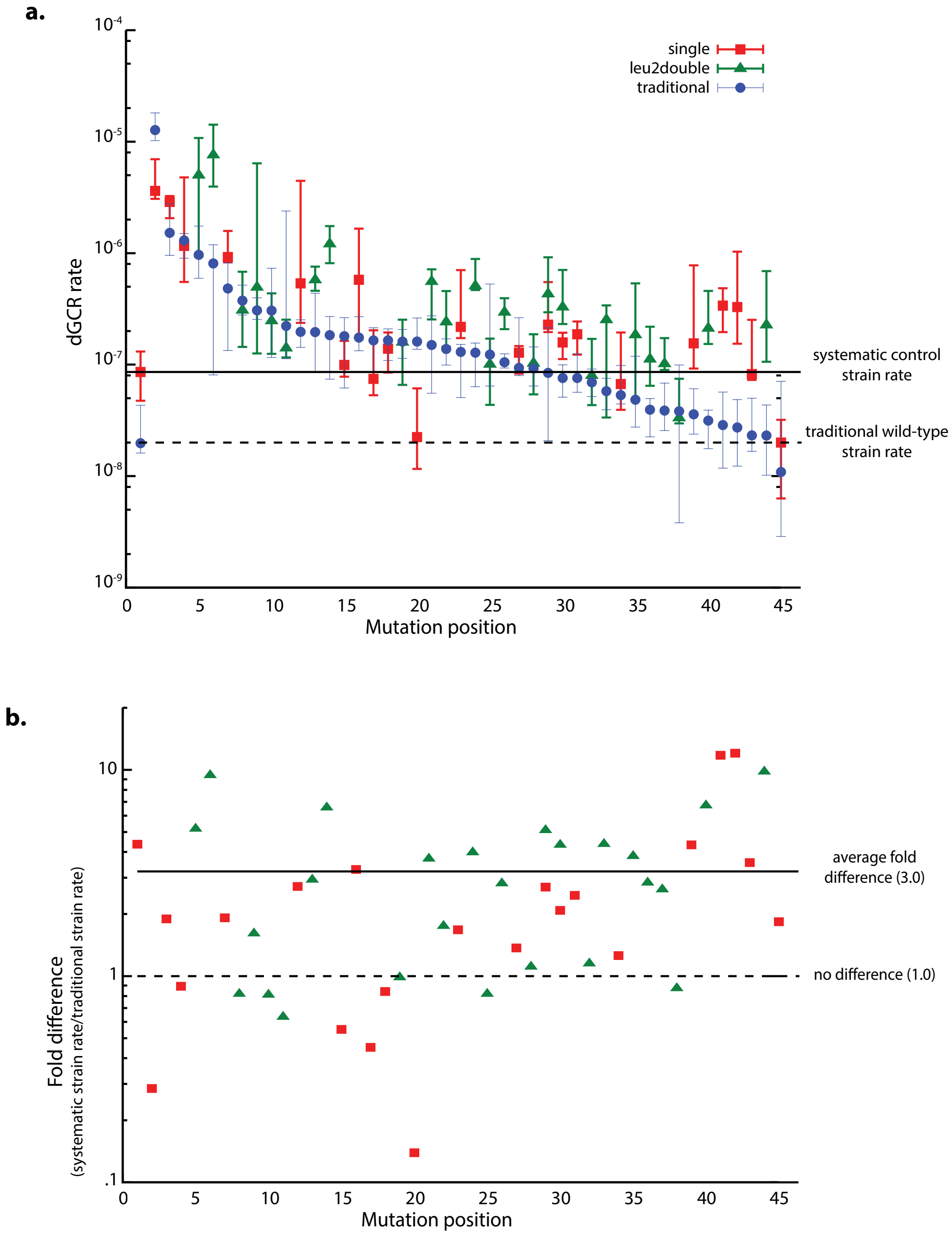

Supplementary Figure 1. dGCR rates for systematically generated strains tend to be higher than for traditionally generated strains. a. The GCR rates for strains with 45 different genotypes are plotted so that strains with the same mutations have the same position along the x-axis. For example, the wild-type strain (or *leu2Δ* control strain) has an x-position of '1'. The GCR rates for the mutant strains constructed in the RDKY6678 background using traditional gene knock out methods[1] are displayed as blue circles with error bars corresponding to the 95% confidence interval. The GCR rates for strains constructed by crossing the dGCR query strain RDKY7635 to the BY4741 mutant collection and isolating appropriate single mutants are displayed as red squares. The GCR rates for strains constructed by crossing a dGCR query strain derived from RDKY7635 containing a mutation of interest *yfgΔ* (Your Favorite Gene) to the BY4741 mutant collection and isolating haploid *leu2Δ yfgΔ* double mutants are displayed as green triangles. The GCR rate for the traditionally constructed wild-type control strain is indicated by the dashed horizontal line, and the GCR rate for the systematically constructed wild-type control strain is indicated by the solid horizontal line. **b.** The fold difference between the GCR rates of the systematically constructed strains and the traditionally constructed strains are plotted. The average fold difference for the 23 single mutant strains (red squares) is 2.7, for the 25 *leu2Δ yfgΔ* double mutant strains (green triangles) is 3.3, and is 3.0 for all 48 systematically generated strains (solid line). No change is indicated by the dashed line. The small but consistent increase may be because the traditionally generated strains have a *can1::hisG* deletion lacking homology to the yeast genome, whereas the systematically generated strains have a *can1::*P_{LEU2}*-NAT* marker that bears a small ~100 bp of *YCLWdelta5* sequence, which has homology to the many Ty element-related sequences in the genome.

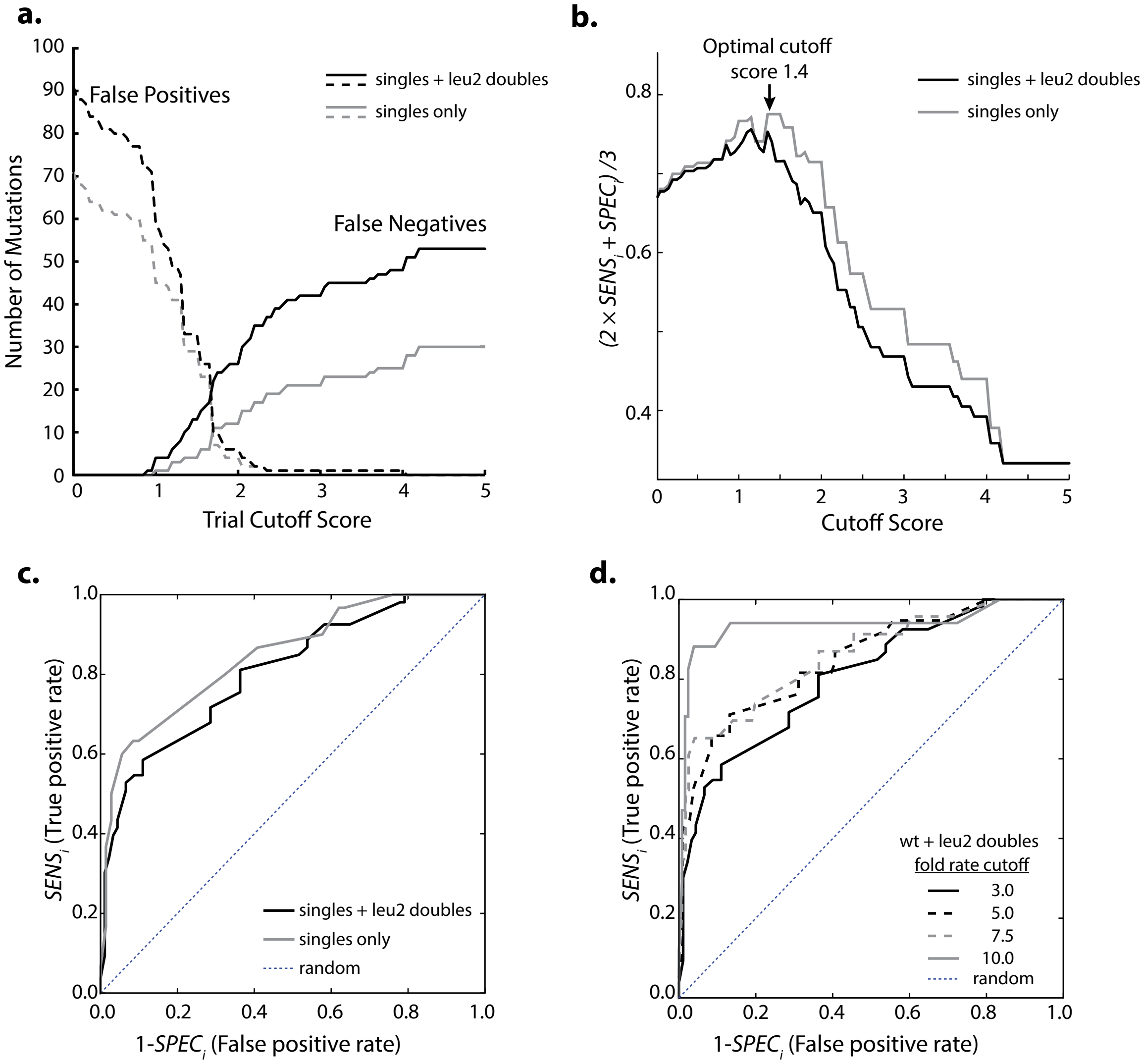

Supplementary Figure 2. Determination of a cutoff score for the dGCR assay. a. The number of incorrectly categorized mutants are plotted as a function of trial patch cutoff scores; strains with GCR rates that are at least 3-fold higher than the control strain are considered to be the "gold-standard" for identifying strains with increased GCR rates. False positives, which have average patch scores above the trial cutoff but do not have increased GCR rates, are displayed with dashed lines. False negatives, which do not have average patch scores above the trial cutoff but do have increased GCR rates, are displayed with solid lines. Grey lines include only data for 101 single mutant strains generated by crossing the wild-type dGCR query strain to the BY4741 mutant collection. Black lines include these 101 strains as well as 43 double mutant strains generated by crossing a dGCR + query mutation strain containing a mutation of interest, *yfgΔ*, to the BY4741 mutant collection and isolating a haploid *leu2Δ yfgΔ* double mutant. The total number of false-positive and false-negative errors can be minimized using a cutoff between 1 and 2. **b.** The weighted sum of the sensitivity and specificity (see Experimental Procedures) plotted as a function of trial cutoff scores reveals a peak around 1.4 for both the 101 single mutant strains (grey) or all 144 mutant strains (black). **c.** The ROC curve reveals that strain scores are a good surrogate for the quantitatively measured rate (compare to dashed random line). The greater area under the curve for the 101 single mutant strains (grey) relative to all 144 mutant strains (black) suggests that the single mutant strain scores are a slightly better predictor than the *leu2Δ yfgΔ* double mutant strain scores. **d.** The increase of the area under the ROC curves using different rate cutoffs as the "gold standard" for increased GCR rates shows, as expected, that the ability of the patch scores to identify strains with increased GCR rates improves for strains with larger increases in GCR rates.

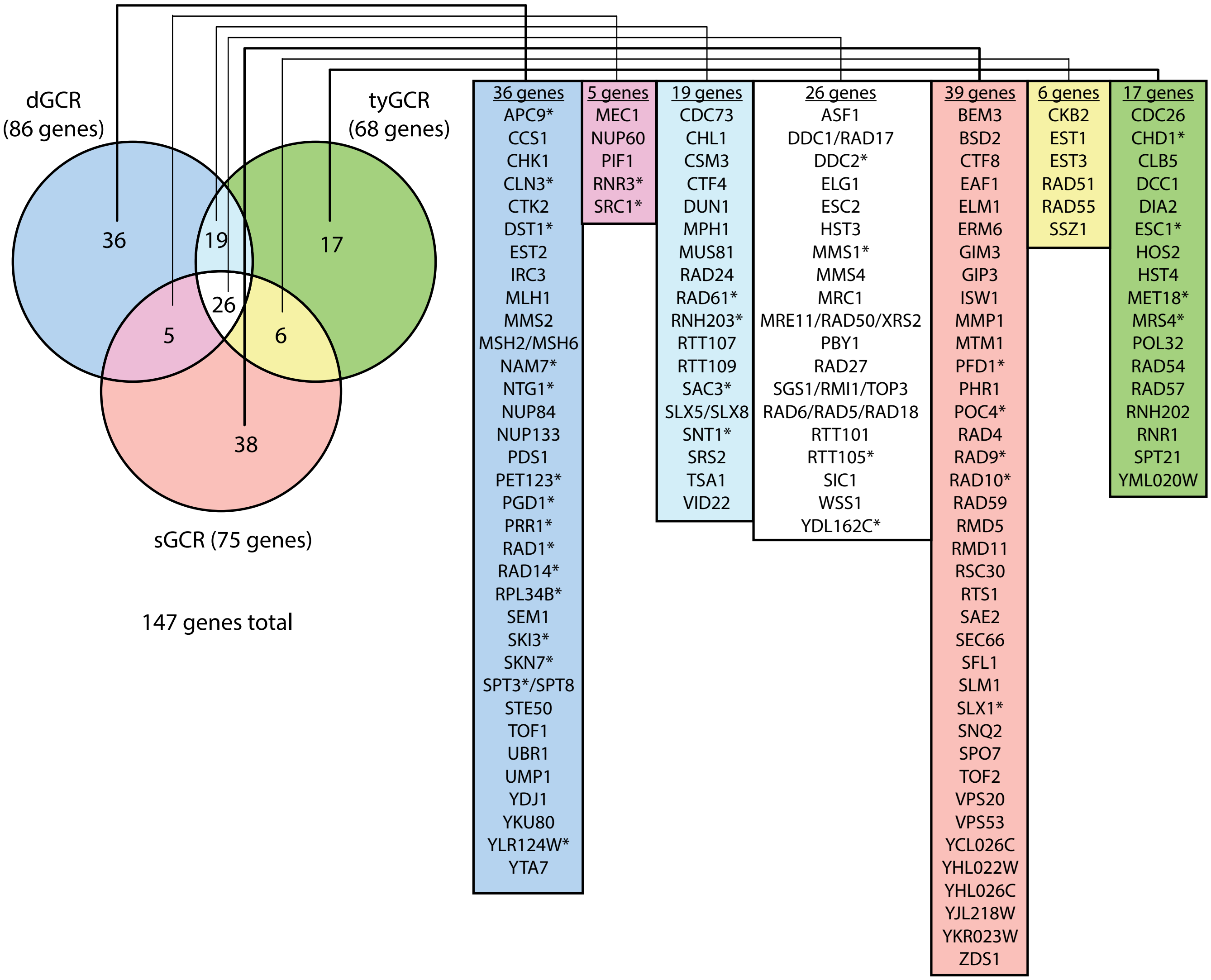

Supplementary Figure 3. Comparison of GIS genes by strain score in the dGCR, sGCR, and tyGCR assays. Venn diagram illustrating the 147 genes suppressing GCRs in the three GCR assays demonstrates that most gene defects were observed in more than one assay. Genes displayed here correspond to only the genes identified by GCR strain scores. Genes that were identified by their GCR strain score but did not increase the GCR rate by at least 3-fold over wild-type in at least one assay, and hence were false positives, are indicated with asterisks (*).

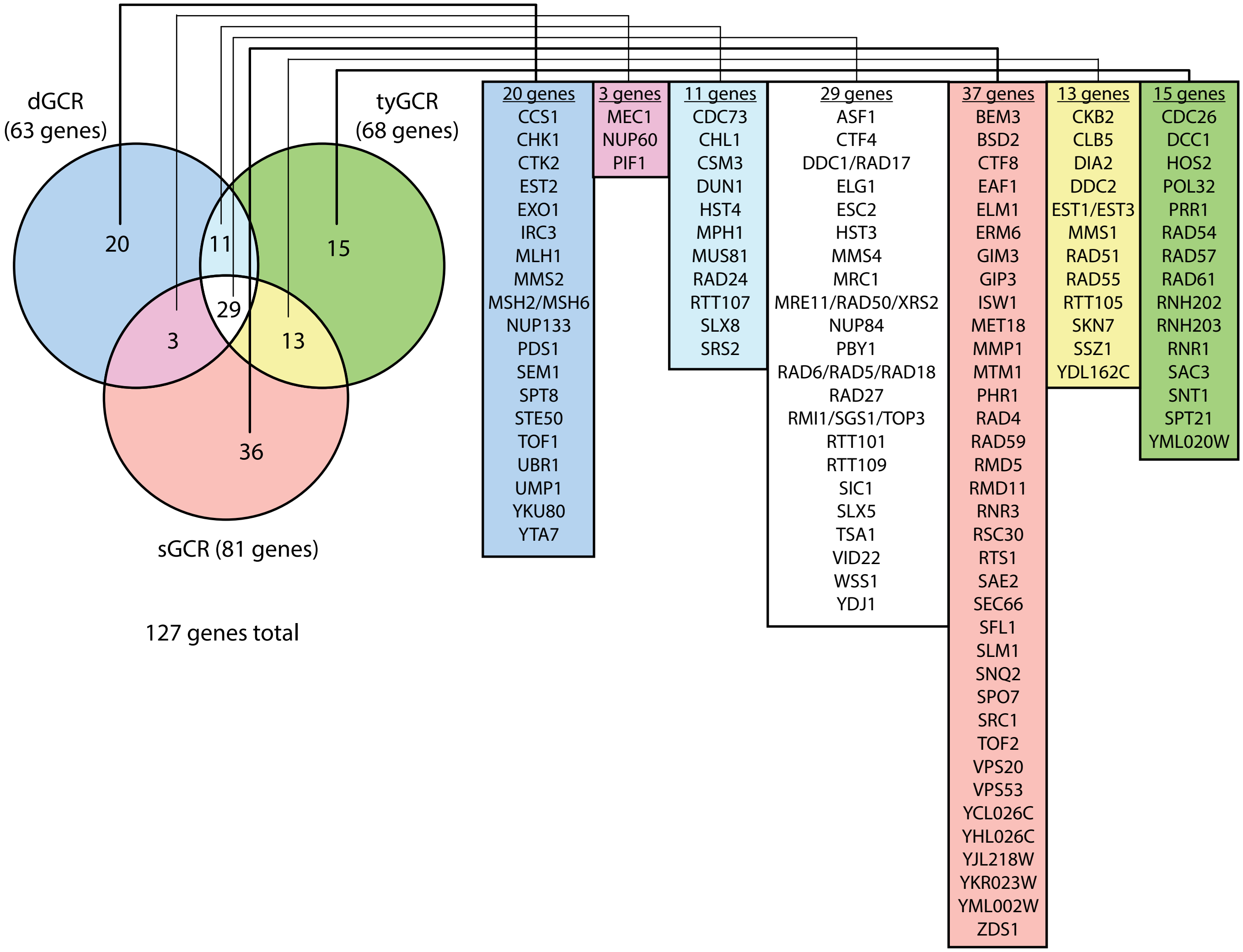

Supplementary Figure 4. Comparison of GIS genes in systematically generated strains in the dGCR, sGCR, and tyGCR assays. Venn diagram illustrating the 126 genes suppressing GCRs in the three GCR assays demonstrates that most genes were observed in more than one assay. This diagram was modified from Supplementary Figure 3 by removing genes corresponding to mutations that did not increase the GCR rate (Supplemental Table 2) by at least 3-fold in at least one assay (i.e. false positives) and by including genes that did increase the GCR rate by at least 3-fold in one assay even if their GCR strain score was below the strain score cutoff score threshold (i.e. false negatives). The decrease of 20 genes relative to that reported in Supplemental Figure 3 reflects the fact that the GCR rates reported (Supplemental Table 2) were to a large extent determined for mutants with GCR strain scores in the range of 1.0-2.0 in order to better derive cutoff scores, but this strain score range is the most difficult to classify using patch tests and strain scores.

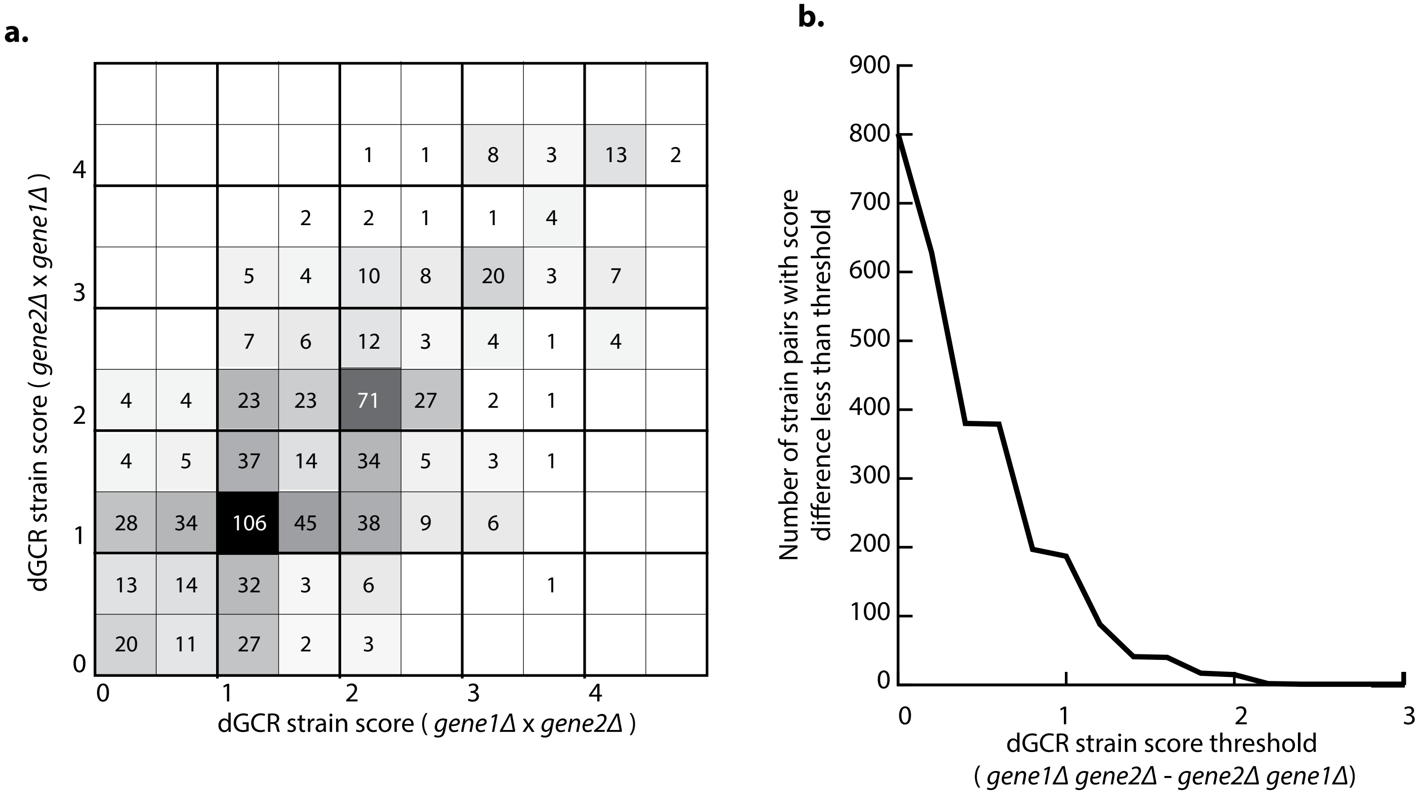

Supplementary Figure 5. Consistency of double mutant scores. a. GCR strain scores for the 801 pairs of double mutants generated in bait × query and query × bait crosses were placed into two-dimensional bins, and the number of pairs in each bin is indicated by the number presented. Most double mutant strains generated in bait × query crosses had a very similar GCR strain score to those generated as in query × bait crosses, so that most pairs fall in bins along the diagonal. **b.** The number of strain pairs with score differences less than a threshold difference is plotted against the threshold difference.

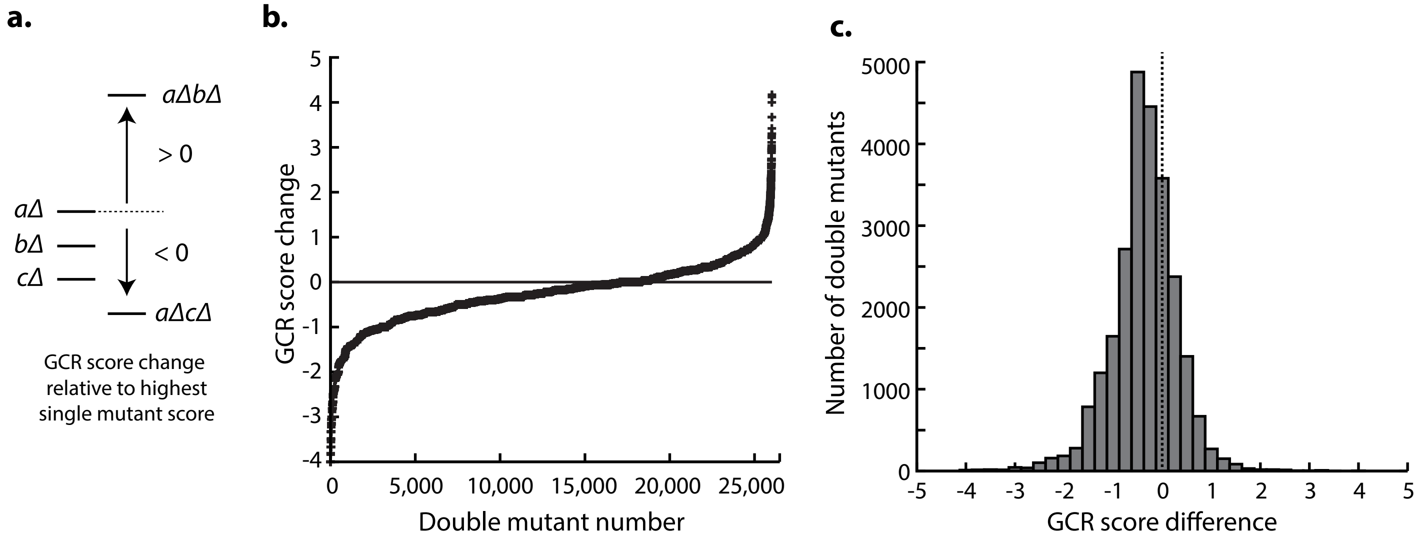

Supplementary Figure 6. Distribution of the double mutant strain scores relative to the strain score of the query single mutants. a. The GCR strain score change of a double mutant strain was determined relative to the higher of the two single mutant GCR strain scores. The score change can be positive (the GCR strain score of the *aΔ bΔ* double mutant is greater than the higher single mutant GCR strain score of *aΔ*) or negative (the GCR strain score of the *aΔ cΔ* double mutant is lower than the higher single mutant GCR strain score of *aΔ*). **b.** GCR strain score change for the entire collection of double mutants, sorted by score change. **c.** The histogram of all GCR strain score changes for the double mutants is centered around zero.

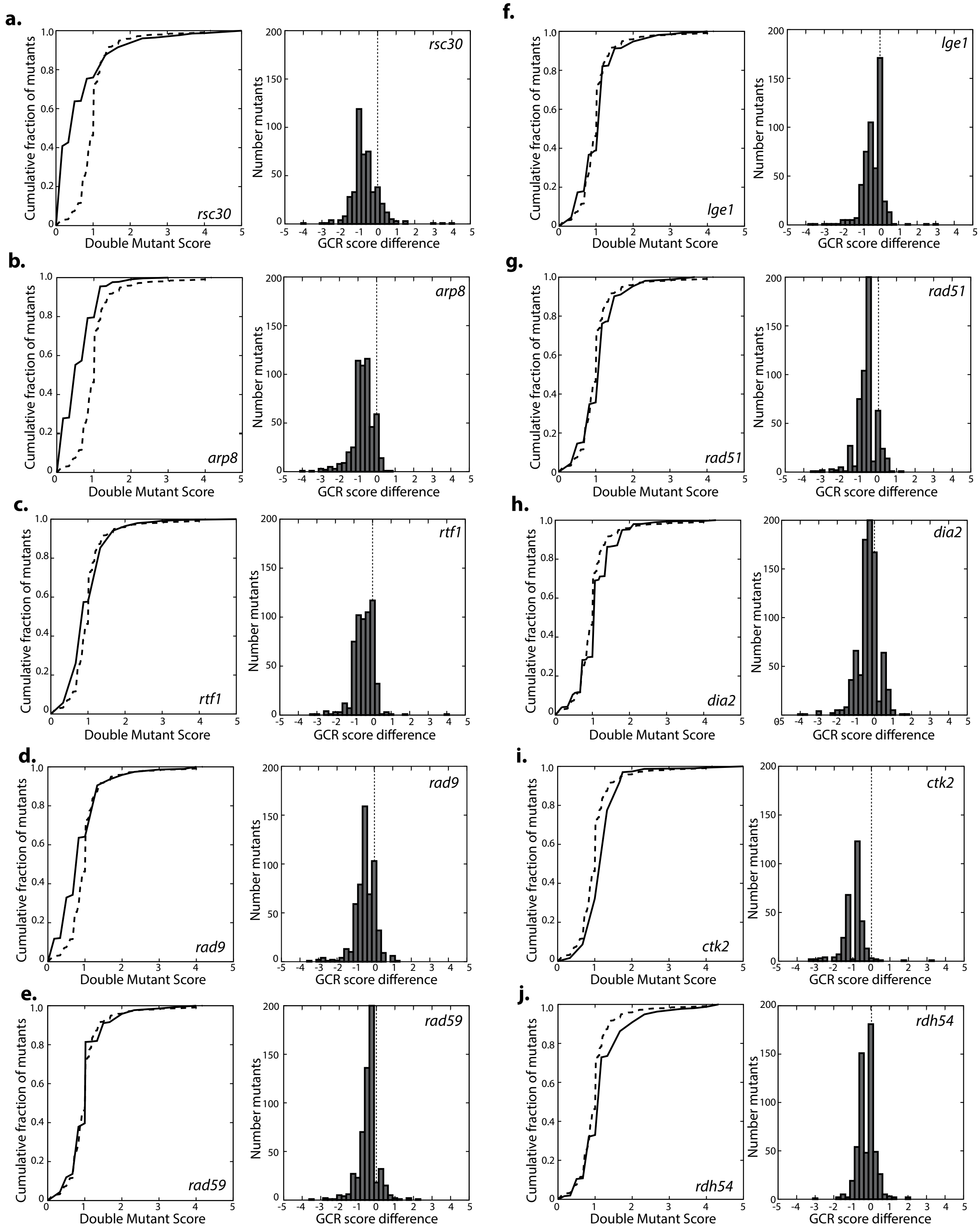

Supplementary Figure 7. Analysis of double mutant strain scores for strains generated in crosses with the *rsc30Δ, arp8Δ, rtf1Δ, rad9Δ, rad59Δ, lge1Δ, rad51Δ, dia2Δ, ctk2Δ,* and *rdh54Δ* query mutants. (Left panels) Plots of the cumulative fraction of mutants (y-axis) with GCR strain scores below the indicated GCR strain scores (x-axis). The data plotted for mutants crossed to strains containing the indicated query mutations are shown by the solid line and the data plotted for mutants crossed to the wild-type strain are shown by the dashed line. **(Right panels)** Histograms of the number of mutations in combination with the query mutations as a function of the score difference, which is the score of the double mutant strain (*aΔ bΔ*) minus the score of the higher of the two single mutant strains (*aΔ* or *bΔ*).

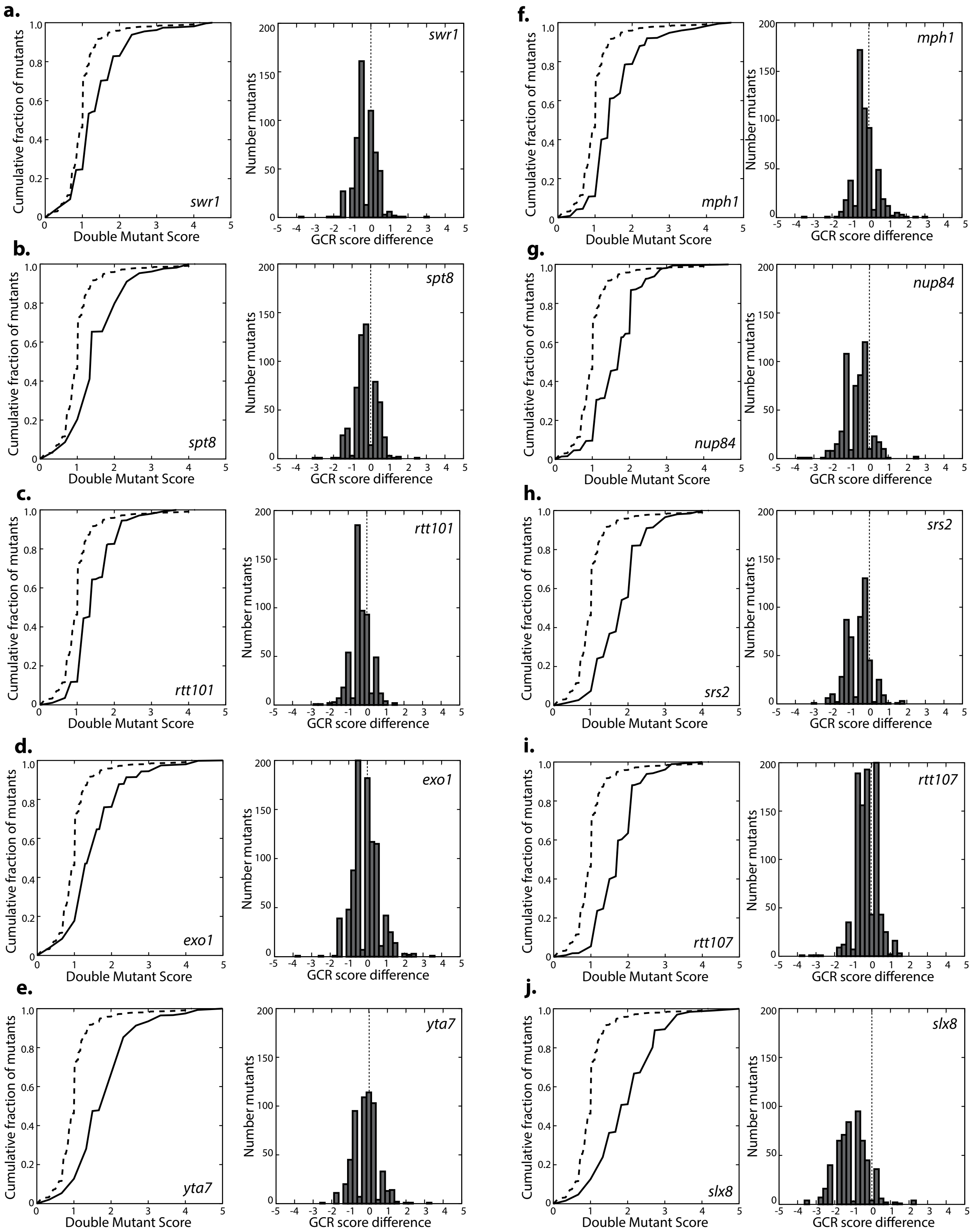

Supplementary Figure 8. Analysis of double mutant strain scores for strains generated in crosses with the *swr1Δ, spt8Δ, rtt101Δ, exo1Δ, yta7Δ, mph1Δ, nup84Δ, srs2Δ, rtt107Δ,* and *slx8Δ* query mutants. Data are plotted as described in Supplementary Figure 7.

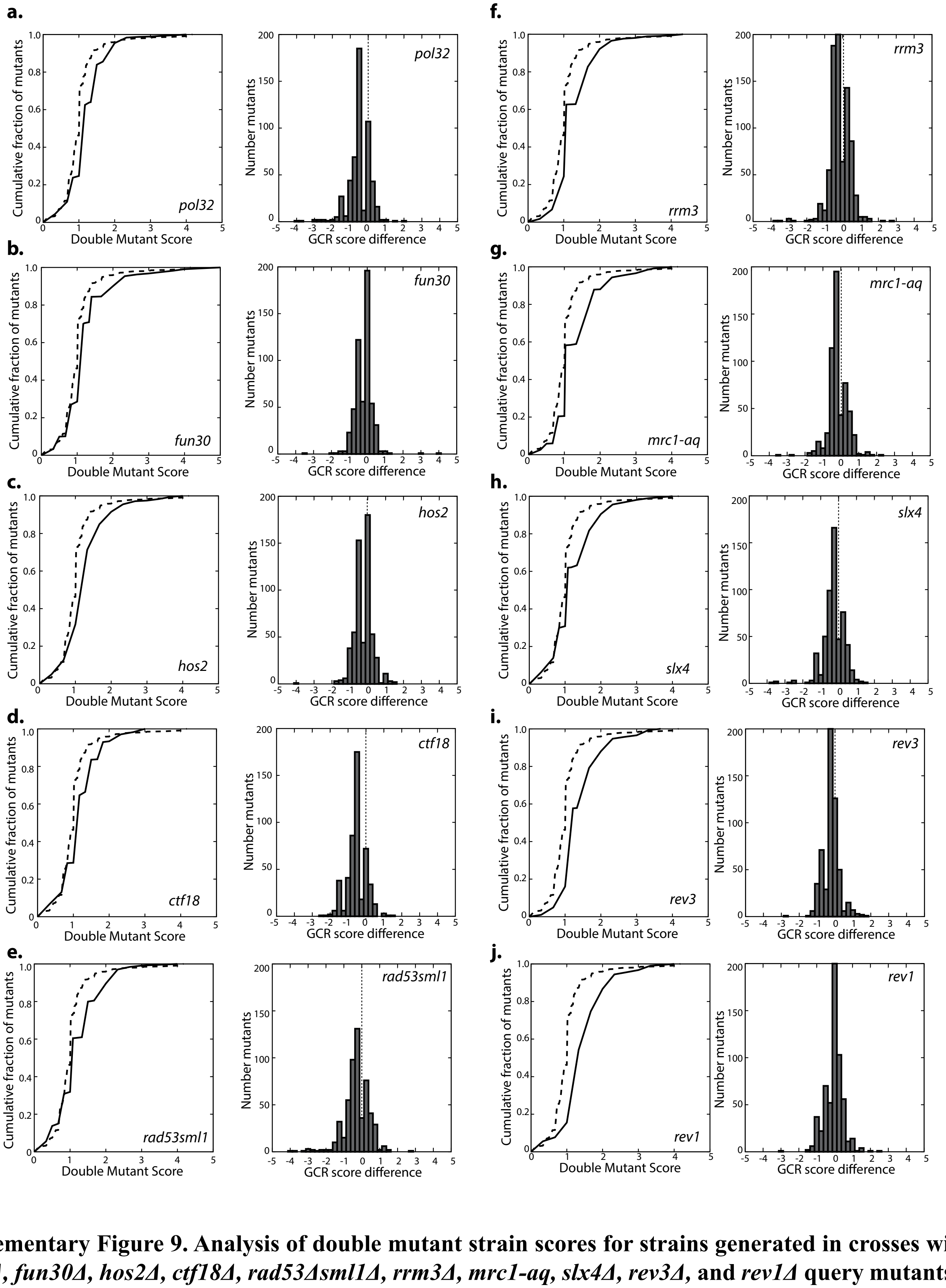

Supplementary Figure 9. Analysis of double mutant strain scores for strains generated in crosses with the *pol32Δ*, *fun30Δ*, *hos2Δ*, *ctf18Δ*, *rad53Δsml1Δ*, *rrm3Δ*, *mrc1-aq*, *slx4Δ*, *rev3Δ*, and *rev1Δ* query mutants. Data are plotted as described in Supplementary Figure 7.

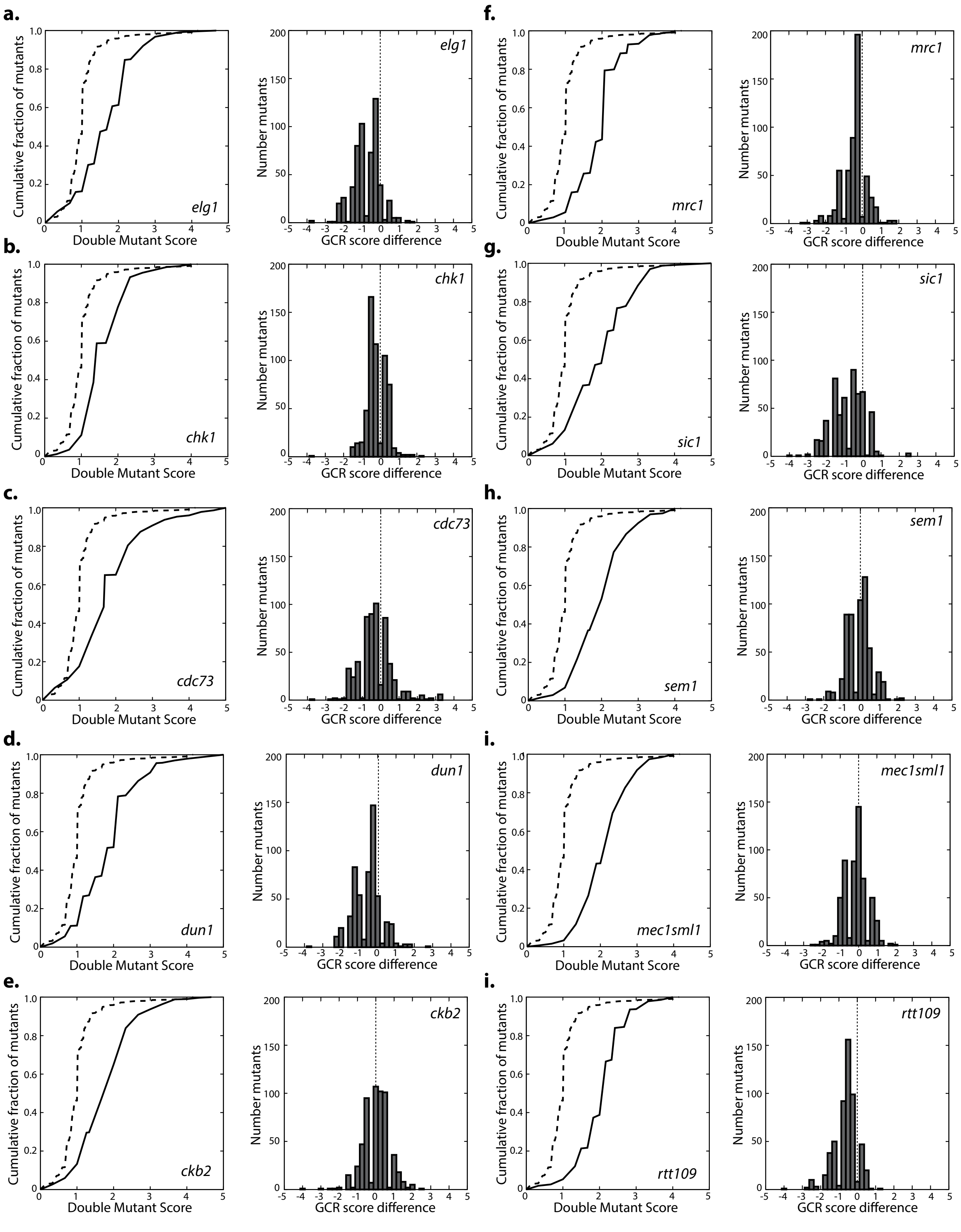

Supplementary Figure 10. Analysis of double mutant strain scores for strains generated in crosses with the *elg1Δ, chk1Δ, cdc73Δ, dun1Δ, ckb2Δ, mrc1Δ, sic1Δ, sem1Δ, mec1Δsml1Δ,* and *rtt109Δ* query mutants. Data are plotted as described in Supplementary Figure 7.

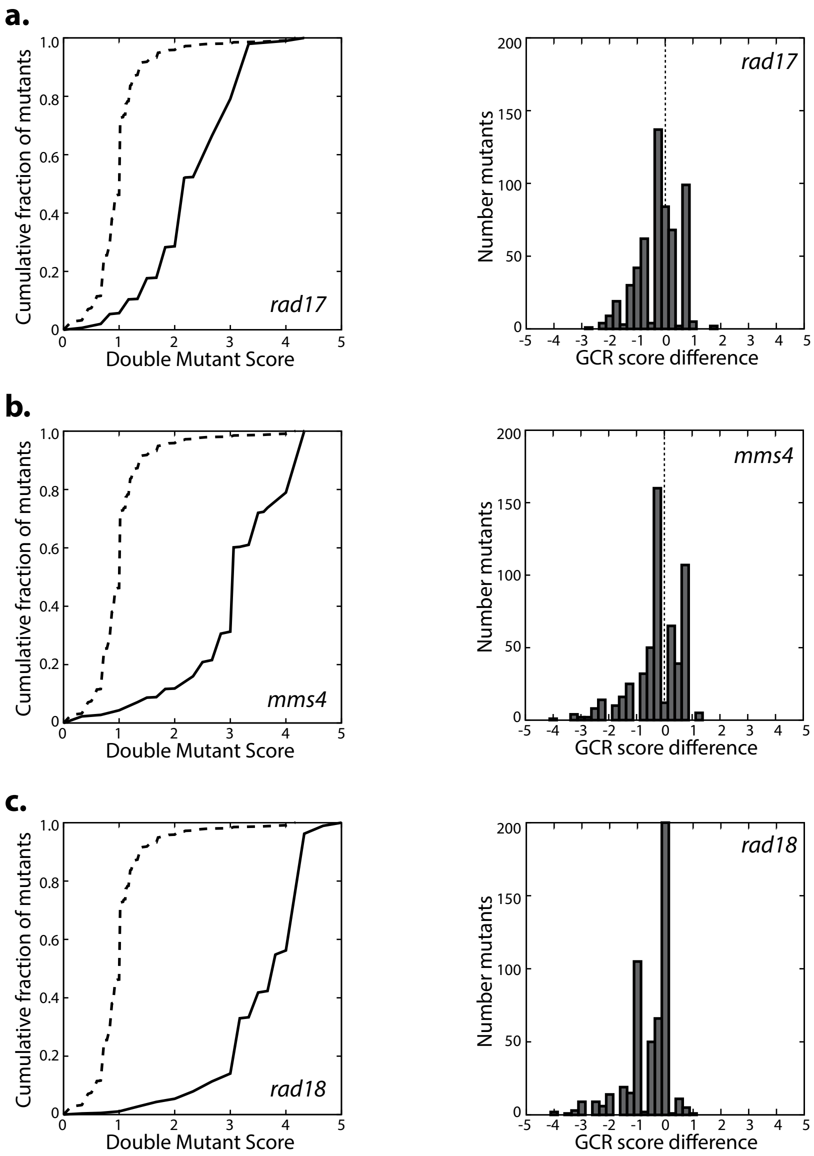

Supplementary Figure 11. Analysis of double mutant strain scores for strains generated in crosses with the *rad17Δ, mms4Δ,* and *rad18Δ* query mutants. Data are plotted as described in Supplementary Figure 7.

a.

Queries (columns) × Baits (rows)

Baits	wt	*srs2*	*rev1*	*rev3*	*rad18**
leu2	0.95	1.67	1.67	1.67	4.00
srs2	2.33	-	1.67	2.00	3.00
rad6	2.33	1.67	2.00	2.33	3.33
ubc13	1.33	2.00	3.00	2.67	3.67
mms2	1.50	1.33	3.00	3.00	3.67
rev1	1.67	2.33	-	1.67	4.00
rev3	1.67	2.00	1.00	-	4.00
rev7	1.33	2.00	1.33	1.33	4.00
*rad5**	3.83	2.00	3.00	3.00	4.00
*rad18**	4.00	2.00	3.00	3.00	-

b.

Queries (columns) × Baits (rows)

Baits	wt	*rev1*	*rev3*	*mph1*
leu2	0.95	1.67	1.67	1.22
rev1	1.67	-	1.67	4.00
rev3	1.67	1.00	-	3.33
rev7	1.33	1.33	1.33	3.33
ubc13	1.33	3.00	2.67	2.67
mms2	1.50	3.00	3.00	1.33
mph1	1.50	3.00	3.00	-

c.

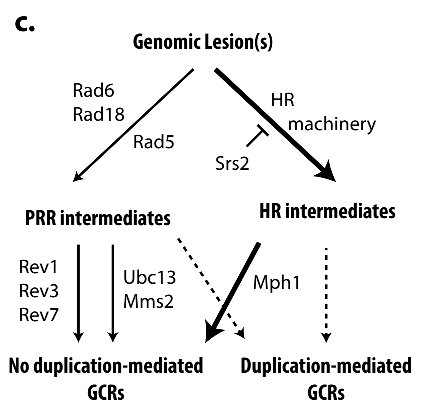

d.

Queries (columns) × Baits (rows)

Baits	wt	*rad17*	*mec1*	*rad53*	*mrc1*	*mrc1-aq*	*rad9*	*chk1*	*dun1*
leu2	0.95	2.19	1.89	1.08	2.07	1.03	0.65	1.42	2.12
rad24	2.17	2.67	2.00	2.00	-	2.00	2.00	2.00	2.00
rad17	2.19	-	3.00	2.67	1.00	3.00	2.00	1.67	1.67
ddc1	2.33	2.00	2.67	0.67	-	2.33	1.33	1.67	2.67
mec3	0.83	1.67	2.67	2.00	1.67	1.67	0.33	1.33	1.67
mec1 sml1	1.89	3.00	-	1.33	0.00	2.00	1.33	1.33	2.00
ddc2 sml1	2.00	3.00	1.67	1.67	1.33	1.33	1.67	1.00	1.67
tel1	1.22	3.33	3.33	1.33	2.00	2.00	1.00	2.00	2.33
rad53 sml1	1.08	1.67	2.33	-	1.00	0.67	1.67	1.33	0.67
rad53-21	1.00	1.83	2.33	-	1.00	2.67	1.00	0.00	-
mrc1	2.07	-	1.00	1.67	-	-	1.33	0.67	2.00
mrc1-aq	1.03	2.67	2.00	1.00	-	-	1.00	1.00	1.67
tof1	1.50	1.67	1.67	-	-	2.00	1.00	0.33	3.00
csm3	1.83	1.67	2.00	1.00	3.00	3.00	1.00	1.67	3.00
rad9	0.65	2.33	1.67	1.00	0.67	0.67	-	0.67	0.33
chk1	1.42	2.67	2.00	1.67	2.33	3.00	0.67	-	1.67
dun1	2.12	1.33	2.00	1.00	2.00	2.00	0.00	1.00	-

e.

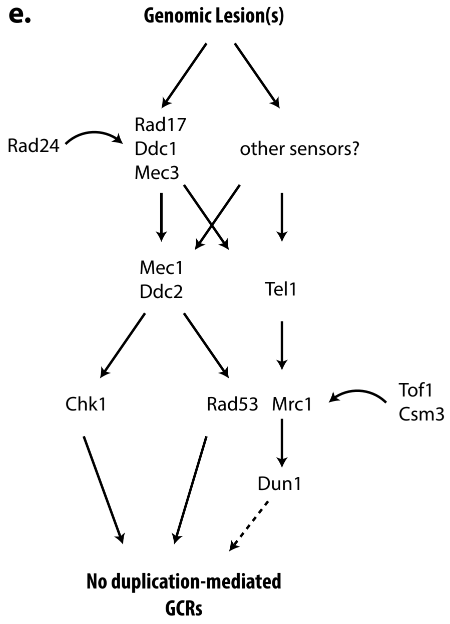

Supplementary Figure 12. Selected genetic interactions between mutations that result in increases in GCRs detected by the dGCR assay. a. Genetic interactions between deletions in post-replication repair (PRR) genes are displayed using GCR strain scores; increases in GCR strain scores in double mutant strains are highlighted with red boxes, and suppressive interactions are highlighted with blue boxes. Defects in the Rev1-Rev3-Rev7 branch and defects in the Ubc13-Mms2 branch cause increases in GCRs, as previously observed in the dGCR assay[2]. Deletion of *RAD18* or *RAD5* causes sufficiently high GCR strain scores that further increases in GCR strain scores in double mutants are difficult to identify; however, partial suppression (blue boxes) of the *rad18Δ* and *rad5Δ* strain scores by *srs2Δ* was detected, as observed previously using quantitative GCR rate tests[2]. **b.** Defects in the Rev1-Rev3-Rev7 and Ubc13-Mms2 PRR branches interact genetically with *mph1Δ* as detected using GCR strain scores and GCR rates (Supplementary Table 7), consistent with the previously observed synergistic interaction between *mph1Δ* and *rad5Δ*[2]. **c.** The model for these genetic interactions argues that PRR plays a role in suppressing GCRs in the dGCR assay by shifting the repair of some lesions from homologous recombination (HR), which can generate GCRs when non-allelic HR targets are involved. Increases in GCRs caused by *mph1Δ* are consistent with the role of Mph1 in preventing aberrant processing of HR intermediates[3]. **d.** Genetic interactions between defects in DNA damage and replication checkpoint genes are displayed as in panel a. Synergy between the deletion of *RAD17* and *mec1Δ, ddc2Δ*, and *tel1Δ* mutations suggests that the Rad17-Ddc1-Mec3 complex signals both the Mec1- and Tel1-dependent checkpoint pathways. Synergy

of *mec1Δ* with *tel1Δ* is consistent with the partial redundancy between *MEC1* and *TEL1*, which encode protein kinases[4]; synergy of *mec1Δ* with defects in Rad17-Ddc1-Mec3 and Rad53 argues that in the absence of Mec1, the Tel1 checkpoint-signaling pathway also utilizes these components. The GCR strain scores for the *rad53Δ* mutations are complicated by the slow growth of *rad53Δ* single and double mutants, which likely leads to a lowered GCR strain score. The synergistic interactions of the checkpoint-deficient but replication-proficient *mrc1-aq* allele indicate that Mrc1-dependent signaling likely involves Mec1-Ddc2 but not Tel1 and that the Rad17-Ddc1-Mec3 complex functions in an alternative pathway. Synergy of *mrc1-aq* with *tof1Δ* and *csm3Δ* could suggest that Tof1-Csm3 might be involved in the signaling pathway, or that more replication damage occurs in *tof1Δ* and *csm3Δ* mutants as Tof1-Csm3 is important for proper localization of Mrc1 to fulfill its roles in DNA replication[5]. In contrast to the effects of mutations in *MRC1*, a deletion of *RAD9* has few increased genetic interactions with other checkpoint mutations (Supplementary Figure 9), suggesting that the replication checkpoint is more important than the general DNA damage checkpoint in suppressing GCRs detected in the dGCR assay. Deletion of the genes encoding the downstream kinases Chk1 and Dun1 have very few interactions with other checkpoint genes, except for the interactions between *dun1Δ* and both *tof1Δ* and *csm3Δ* mutations. **e.** A model consistent with these observed interactions in the suppression of GCRs detected in the dGCR assay is shown.

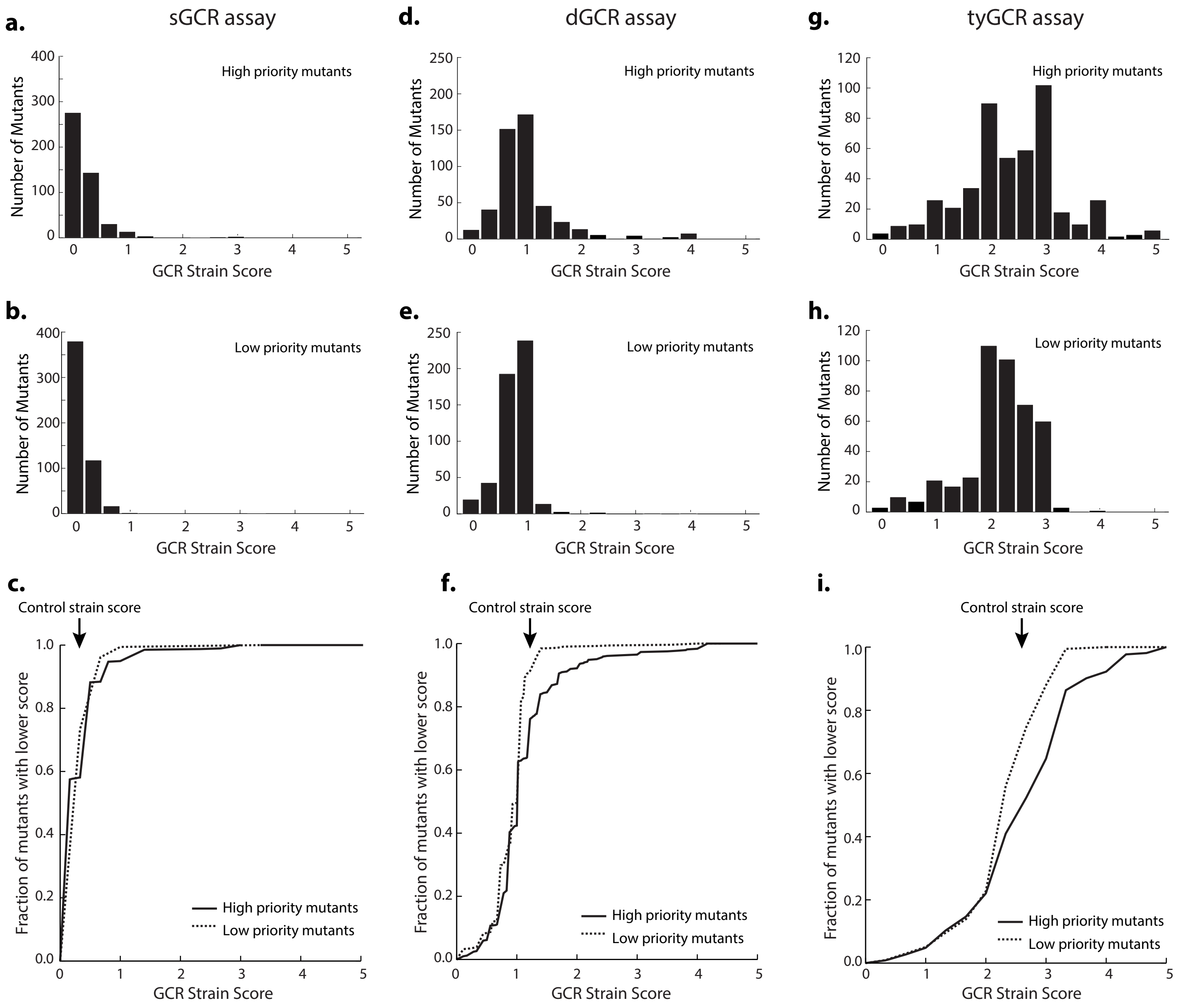

Supplementary Figure 13. Distribution of GCR strain scores for the "high priority" and "low priority" sets of single mutations. Histograms of the number of mutant strains as a function of the average GCR strain score reveals that a greater number of mutations in the high-priority set (Supplementary Data 1) cause higher GCR strain scores in the sGCR assay **(panel a)**, dGCR assay **(panel d)**, and tyGCR assay **(panel g)** compared to the low priority set of mutations (Supplementary Data 1) in the sGCR assay **(panel b)**, dGCR assay **(panel e)**, and tyGCR assay **(panel h)**. The GCR strain scores also were converted into monotonically increasing cumulative distribution functions for the sGCR assay **(panel c)**, dGCR assay **(panel f)**, and tyGCR assay **(panel i)**. The deviation of these curves from a step function reflects both stochastic numbers of papillae in each patch as well as genetic influences on the GCR rate and growth rate of the cells. Notably, mutations in the high-priority genes, which were predicted to more likely cause increased GCR rates, show a stronger deviation from a step function for all three assays.

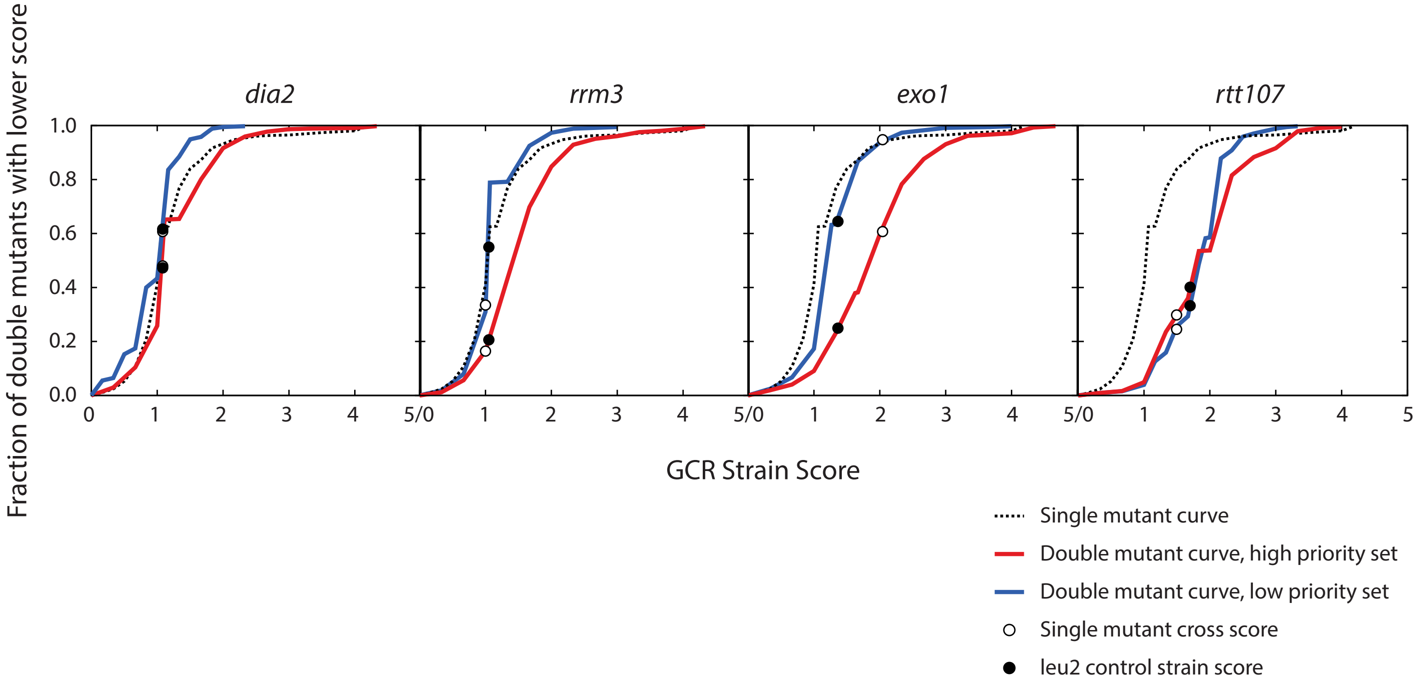

Supplementary Figure 14. Distribution of dGCR strain scores for the "high priority" and "low priority" sets of mutations when crossed to the *dia2Δ, rrm3Δ, exo1Δ,* and *rtt107Δ* mutant query strains. The dGCR strain scores are displayed using a cumulative distribution function of the fraction of double mutants with a lower strain score for the high- (red line) and the low- (blue line) priority sets of bait mutations (Supplementary Data 1), as compared to the single mutant distribution for all mutations (dashed black line). For each of these mutants, the high-priority set shows the greatest deviation from a step function, indicating a larger number of genetic interactions with bait mutations.

Supplementary Table 1. Differing effects of some mutations in the three variant GCR assays.

category	*genotype*	*dGCR rate*	*tyGCR rate*	*sGCR rate*
Control strain				
	leu2Δ	8.59×10^{-8} (1)	3.21×10^{-7} (1)	4.00×10^{-9} (1)
Increased in all				
	vid22Δ	1.97×10^{-6} (22.9)	2.45×10^{-6} (7.6)	2.80×10^{-8} (5.2)
	ydj1Δ	1.39×10^{-6} (16.2)	1.51×10^{-6} (4.7)	2.47×10^{-8} (6.2)
	rtt109Δ	1.21×10^{-6} (14.1)	7.57×10^{-6} (23.6)	5.01×10^{-8} (12.5)
	tsa1Δ	1.16×10^{-6} (13.5)	6.61×10^{-6} (20.6)	6.74×10^{-8} (16.9)
	slx5Δ	9.24×10^{-7} (10.8)	5.69×10^{-6} (17.7)	1.56×10^{-7} (39.0)
	ctf4Δ	4.71×10^{-7} (5.7)	2.69×10^{-6} (8.4)	1.37×10^{-8} (3.4)
	nup84Δ	3.18×10^{-7} (3.7)	7.31×10^{-6} (22.8)	3.99×10^{-8} (10.0)
Increased in dGCR+tyGCR				
	csm3Δ	3.18×10^{-7} (3.7)	1.34×10^{-6} (4.2)	6.74×10^{-9} (1.7)
	srs2Δ	5.10×10^{-7} (5.9)	1.97×10^{-6} (6.1)	1.10×10^{-9} (0.3)
	hst4Δ	5.31×10^{-7} (6.2)	9.51×10^{-7} (3.0)	4.98×10^{-9} (1.2)
Increased in tyGCR+sGCR				
	dia2Δ	2.15×10^{-7} (2.5)	5.81×10^{-6} (18.1)	3.11×10^{-8} (7.8)
	ssz1Δ	2.06×10^{-7} (2.4)	5.00×10^{-6} (15.6)	1.90×10^{-7} (47.5)
	clb5Δ	1.79×10^{-7} (2.1)	2.45×10^{-6} (7.6)	1.26×10^{-8} (3.5)
	rad51Δ	2.27×10^{-7} (2.6)	1.60×10^{-6} (5.0)	9.26×10^{-8} (23.2)
	skn7Δ	1.52×10^{-7} (1.8)	1.23×10^{-6} (3.8)	2.50×10^{-8} (6.3)
Increased in dGCR				
	nup133Δ	1.21×10^{-6} (14.1)	5.56×10^{-10} (0.0)	2.05×10^{-9} (0.5)
	msh2Δ	5.76×10^{-7} (6.7)	8.78×10^{-7} (2.7)	2.13×10^{-9} (0.5)
	exo1Δ	4.31×10^{-7} (5.0)	4.41×10^{-7} (1.4)	9.30×10^{-9} (2.3)
Increased in tyGCR				
	prr1Δ	1.67×10^{-7} (1.9)	1.36×10^{-6} (4.2)	2.42×10^{-9} (0.6)
	pol32Δ	2.12×10^{-7} (2.5)	1.08×10^{-6} (3.4)	4.92×10^{-9} (1.2)
Increased in sGCR				
	sae2Δ	1.39×10^{-7} (1.6)	6.66×10^{-7} (2.1)	5.17×10^{-8} (12.9)
	met18Δ	6.77×10^{-8} (0.8)	4.57×10^{-7} (1.4)	1.57×10^{-8} (3.9)
No significant increase				
	rad9Δ	3.34×10^{-8} (0.4)	5.25×10^{-7} (1.6)	7.17×10^{-9} (2.0)
	poc4Δ	8.73×10^{-8} (1.0)	1.22×10^{-7} (0.4)	3.35×10^{-9} (0.8)
	pfd1Δ	1.95×10^{-7} (2.3)	9.16×10^{-7} (2.9)	1.64×10^{-9} (0.4)
	cln3Δ	1.83×10^{-7} (2.1)	6.70×10^{-7} (2.1)	5.69×10^{-9} (1.4)
	mrs4Δ	1.60×10^{-7} (1.9)	4.35×10^{-7} (1.4)	2.27×10^{-9} (0.6)
	esc1Δ	1.28×10^{-7} (1.5)	4.19×10^{-7} (1.3)	4.24×10^{-9} (1.1)
	rad10Δ	9.93×10^{-8} (1.2)	4.95×10^{-7} (1.5)	2.00×10^{-9} (0.5)
	slx1Δ	8.24×10^{-8} (1.0)	2.75×10^{-7} (0.9)	1.81×10^{-9} (0.5)
	chd1Δ	7.80×10^{-8} (0.9)	6.40×10^{-7}(2.0)	3.90×10^{-9} (1.0)
	rad52Δ	2.00×10^{-8} (0.2)	8.31×10^{-8} (0.3)	7.00×10^{-9} (1.8)

Supplementary Table 2. Single mutant GCR rates in the systematically generated dGCR strains.

genotype	Strains from wild-type query cross		*leu2* strains from mutant query cross	
	GCR strain score	Can^R $5FOA^R$ rate (fold increase)	GCR strain score	Can^R $5FOA^R$ rate (fold increase)
control (*leu2*)	0.94	8.59×10^{-8} (1)	-	-
rmi1	4.2	3.62×10^{-6} (42.1)	-	-
wss1	4.2	4.54×10^{-6} (52.8)	-	-
mre11	4.0	2.88×10^{-6} (33.5)	-	-
rad18	4.0	-	3.8	7.62×10^{-6} (88.7)
rad50	4.0	1.97×10^{-6} (22.9)	-	-
xrs2	4.0	1.82×10^{-6} (21.2)	-	-
rpl34b	4.0	6.75×10^{-8} (0.8)	-	-
tsa1	3.7	1.16×10^{-6} (13.5)	-	-
vid22	3.6	1.97×10^{-6} (22.9)	-	-
pby1	3.0	1.15×10^{-6} (13.4)	-	-
slx5	3.0	9.24×10^{-7} (10.8)	-	-
mms4	2.7	-	3.1	5.81×10^{-7} (6.8)
ydj1	2.6	1.39×10^{-6} (16.2)	-	-
hst3	2.5	4.80×10^{-7} (5.6)	-	-
ddc1	2.3	3.58×10^{-7} (4.2)	-	-
pds1	2.3	3.12×10^{-6} (36.3)	-	-
srs2	2.3	-	2.1	5.10×10^{-7} (5.9)
ylr124w	2.3	1.04×10^{-7} (1.2)	-	-
msh2	2.2	5.76×10^{-7} (6.7)	-	-
nup84	2.2	-	2.1	3.18×10^{-7} (3.7)
rad24	2.2	5.36×10^{-7} (6.2)	-	-
ctf4	2.0	4.71×10^{-7} (5.5)	-	-
ddc2 sml1	2.0	2.39×10^{-7} (2.8)	-	-
elg1	2.0	-	2.2	2.42×10^{-7} (2.8)
exo1	2.0	2.28×10^{-7} (2.7)	1.3	4.31×10^{-7} (5.0)
nup133	2.0	1.21×10^{-6} (14.1)	-	-
nup60	2.0	5.51×10^{-7} (6.4)	-	-
cln3	1.8	1.83×10^{-7} (2.1)	-	-
csm3	1.8	3.18×10^{-7} (3.7)	-	-
prr1	1.8	1.67×10^{-7} (1.9)	-	-
rad17	1.8	-	2.2	5.70×10^{-7} (6.6)
sic1	1.8	-	2.4	8.99×10^{-7} (10.5)
skn7	1.8	1.52×10^{-7} (1.8)	-	-
cdc73	1.7	3.71×10^{-7} (4.3)	1.7	7.54×10^{-7} (8.8)
chl1	1.7	2.91×10^{-7} (3.4)	-	-
dst1	1.7	1.16×10^{-7} (1.4)	-	-
est2	1.7	6.79×10^{-7} (7.9)	-	-
irc3	1.7	7.04×10^{-7} (8.2)	-	-
mms1	1.7	2.18×10^{-7} (2.5)	-	-
nam7	1.7	1.10×10^{-7} (1.3)	-	-
pet123	1.7	1.45×10^{-7} (1.7)	-	-
rad1	1.7	1.27×10^{-7} (1.5)	-	-
rad14	1.7	7.56×10^{-8} (0.9)	-	-
rad61	1.7	1.16×10^{-7} (1.4)	-	-
rev1	1.7	1.44×10^{-7} (1.7)	1.0	4.19×10^{-7} (4.9)
rev3	1.7	1.58×10^{-7} (1.8)	1.2	3.29×10^{-7} (3.8)
rnh203	1.7	1.73×10^{-7} (2.0)	-	-

rnr3	1.7	9.40×10^{-8} (1.1)	-	-
rtt105	1.7	1.76×10^{-7} (2.0)	-	-
ski3	1.7	1.29×10^{-7} (1.5)	-	-
spt3	1.7	2.33×10^{-7} (2.7)	-	-
spt8	1.7	-	1.4	7.30×10^{-7} (8.5)
src1	1.7	9.09×10^{-8} (1.1)	-	-
yku80	1.7	3.29×10^{-7} (3.8)	-	-
ydl162c	1.7	1.41×10^{-7} (1.6)	-	-
apc9	1.5	4.87×10^{-8} (0.6)	-	-
ckb2	1.5	3.92×10^{-7} (4.6)	1.3	7.47×10^{-7} (8.7)
hos2	1.5	1.02×10^{-7} (1.2)	1.0	1.34×10^{-6} (15.6)
mph1	1.5	-	1.4	2.96×10^{-7} (3.5)
ntg1	1.5	5.24×10^{-8} (0.6)	-	-
pgd1	1.5	1.14×10^{-7} (1.3)	-	-
rtt107	1.5	-	1.7	4.95×10^{-7} (5.8)
sac3	1.5	9.01×10^{-8} (1.0)	-	-
slx8	1.5	-	2.7	5.01×10^{-6} (58.3)
snt1	1.5	2.08×10^{-7} (2.4)	-	-
ump1	1.5	2.89×10^{-7} (3.3)	-	-
mrc1	1.4	-	2.0	3.08×10^{-7} (3.6)
apn1	1.3	1.28×10^{-7} (1.5)	-	-
apn2	1.3	7.70×10^{-8} (0.9)	-	-
cac2	1.3	1.07×10^{-7} (1.3)	-	-
chk1	1.3	-	1.4	5.76×10^{-7} (6.7)
ctf18	1.3	-	1.3	1.41×10^{-7} (1.6)
dun1	1.3	-	2.1	1.59×10^{-7} (1.9)
lif1	1.3	9.92×10^{-8} (1.2)	-	-
mad2	1.3	9.99×10^{-8} (1.2)	-	-
mlh3	1.3	7.07×10^{-8} (0.8)	-	-
nup120	1.3	5.88×10^{-7} (6.9)	-	-
rad10	1.3	9.93×10^{-8} (1.2)	-	-
rnh202	1.3	1.85×10^{-7} (2.2)	-	-
rtt109	1.3	-	2.4	1.21×10^{-6} (14.1)
sae2	1.3	1.39×10^{-7} (1.6)	-	-
sem1	1.3	-	1.6	4.90×10^{-7} (5.7)
shu2	1.3	1.44×10^{-7} (1.7)	-	-
ssz1	1.3	2.06×10^{-7} (2.4)	-	-
ycl036w	1.3	9.83×10^{-8} (1.1)	-	-
yku70	1.3	6.70×10^{-8} (0.8)	-	-
yta7	1.3	-	1.6	7.27×10^{-7} (9.4)
ctk2	1.2	-	1.8	5.26×10^{-8} (0.6)
dia2	1.2	-	1.2	2.15×10^{-7} (2.5)
fun30	1.2	-	1.0	4.40×10^{-8} (0.5)
mec1 sml1	1.2	-	1.9	5.57×10^{-7} (6.5)
met18	1.2	6.77×10^{-8} (0.8)	-	-
rad59	1.2	-	1.0	8.00×10^{-8} (0.9)
rdh54	1.2	-	1.0	9.17×10^{-8} (1.1)
rnh201	1.2	2.86×10^{-7} (3.3)	-	-
tel1	1.2	3.38×10^{-7} (3.9)	-	-
vma8	1.2	5.10×10^{-8} (0.6)	-	-
mrs4	1.1	1.60×10^{-7} (1.9)	-	-
chd1	1.0	7.80×10^{-8} (0.9)	-	-
ckb1	1.0	2.14×10^{-7} (2.5)	-	-
clb5	1.0	1.79×10^{-7} (2.1)	-	-
dnl4	1.0	1.87×10^{-7} (2.2)	-	-

esc1	1.0	1.28×10^{-7} (1.5)	-	-
hst4	1.0	5.31×10^{-7} (6.2)	-	-
lge1	1.0	-	1.0	1.12×10^{-7} (1.3)
poc4	1.0	8.73×10^{-8} (1.0)	-	-
pol32	1.0	-	1.3	2.12×10^{-7} (2.5)
rad30	1.0	7.45×10^{-8} (0.9)	-	-
rad51	1.0	-	1.3	2.27×10^{-7} (2.6)
rad52	1.0	2.00×10^{-8} (0.23)	-	-
rrm3	1.0	-	1.1	1.02×10^{-7} (1.2)
rtt101	1.0	-	1.5	2.53×10^{-7} (3.0)
slx1	1.0	8.24×10^{-8} (1.0)	-	-
slx4	1.0	-	1.1	1.03×10^{-7} (1.2)
mrc1-aq	0.9	-	1.0	1.01×10^{-7} (1.2)
hur1	0.83	3.74×10^{-8} (0.4)	-	-
mec3	0.83	2.24×10^{-8} (0.3)	-	-
psy3	0.83	1.55×10^{-7} (1.8)	-	-
rad54	0.83	1.31×10^{-7} (1.5)	-	-
rtf1	0.83	-	0.9	3.12×10^{-7} (3.6)
set2	0.83	1.62×10^{-7} (1.9)	-	-
swr1	0.83	-	1.3	1.32×10^{-7} (1.7)
arp8	0.67	-	0.7	1.85×10^{-7} (2.2)
pfd1	0.67	1.95×10^{-7} (2.3)	-	-
lsm7	0.50	5.86×10^{-8} (0.7)	-	-
rad9	0.50	-	0.6	3.34×10^{-8} (0.4)
rad53 sml1	0.50	-	1.1	2.48×10^{-7} (2.9)
rsc30	0.50	-	0.3	2.45×10^{-7} (2.9)
dot1	0.33	7.10×10^{-8} (0.83)	-	-
eaf7	0.33	9.93×10^{-8} (1.2)	-	-
cdh1	0.17	1.75×10^{-9} (0.02)	-	-
dep1	0.17	4.19×10^{-8} (0.50)	-	-
rsc2	0.10	1.33×10^{-9} (0.02)	-	-
clb2	0.0	3.13×10^{-8} (0.4)	-	-
pop2	0.0	8.28×10^{-9} (0.10)	-	-
rpd3	0.0	3.24×10^{-8} (0.4)	-	-
ypt6	0.0	8.44×10^{-8} (1.0)	-	-

Supplementary Table 3. dGCR rates of single mutations determined using traditionally generated strains.

Mutation	*GCR Strain Score**	*Rate (fold over wild-type)*	*Reference*
wild-type	1.0	1.97×10^{-8} (1)	13
rmi1Δ	4.2	1.27×10^{-5} (645)	13
esc2Δ	4.0	1.07×10^{-5} (543)	13
mre11Δ	4.0	1.52×10^{-6} (77)	13
rad18Δ	4.0 (3.8)	8.08×10^{-7} (41)	24
sgs1Δ	4.0	1.93×10^{-6} (98)	13
top3Δ	4.0	2.14×10^{-6} (107)	13
rad5Δ	3.8	3.78×10^{-7} (19)	24
tsa1Δ	3.7	1.30×10^{-6} (66)	24
rad27Δ	3.5	2.78×10^{-6} (141)	13
mus81Δ	3.0	2.51×10^{-7} (13)	13
pif1Δ	3.0	3.61×10^{-7} (18)	24
slx5Δ	3.0	4.82×10^{-7} (24)	13
rad6Δ	2.3	6.03×10^{-7} (31)	13
srs2Δ	2.3 (2.1)	1.28×10^{-7} (6.4)	13
msh2Δ	2.2	1.75×10^{-7} (8.9)	13
rad24Δ	2.2	1.97×10^{-7} (10)	13
exo1Δ	2.0	8.44×10^{-8} (4.3)	13
mlh1Δ	2.0	3.85×10^{-8} (2.0)	13
msh6Δ	1.8	2.10×10^{-7} (11)	13
rev3Δ	1.7	7.59×10^{-8} (3.9)	24
yku80Δ	1.7	2.73×10^{-8} (1.4)	20
asf1Δ	1.5	2.89×10^{-7} (14.7)	13
mms2Δ	1.5	2.47×10^{-7} (12.5)	24
mph1Δ	1.5	1.05×10^{-7} (5.3)	13
rtt107Δ	1.5 (1.7)	3.07×10^{-7} (15.6)	13
slx8Δ	1.5	9.65×10^{-7} (49.0)	13
tof1Δ	1.5	4.25×10^{-7} (21.6)	13
ubr1Δ	1.5	1.06×10^{-7} (5.4)	24
dun1Δ	1.3 (2.1)	1.61×10^{-7} (8.2)	13
chk1Δ	1.3 (1.4)	1.96×10^{-7} (9.9)	13
rad10Δ	1.3	1.80×10^{-7} (9.1)	13
rtt109Δ	1.3 (2.4)	1.84×10^{-7} (9.3)	13
sae2Δ	1.3	1.65×10^{-7} (8.4)	13
ubc13Δ	1.3	2.06×10^{-7} (10.5)	24
yku70Δ	1.3	5.33×10^{-8} (2.7)	20
hrq1Δ	1.2	6.32×10^{-8} (3.2)	24
rad59Δ	1.2 (1.0)	6.94×10^{-8} (3.5)	13
tel1Δ	1.2	2.87×10^{-8} (1.5)	13
est3Δ	1.1	1.85×10^{-8} (0.9)	20
bre1Δ	1.0	4.89×10^{-8} (2.4)	24
ctf18Δ	1.0 (1.3)	2.22×10^{-7} (11.3)	13
lig4Δ	1.0	2.87×10^{-8} (1.5)	20
lge1Δ	1.0 (1.0)	3.94×10^{-8} (2.0)	24
msh3Δ	1.0	3.67×10^{-8} (1.9)	13
pol32Δ	1.0 (1.3)	3.15×10^{-8} (1.6)	13
rad30Δ	1.0	1.65×10^{-7} (8.4)	24
rad52Δ	1.0	1.09×10^{-8} (0.6)	13
rad51Δ	1.0 (1.3)	2.31×10^{-8} (1.2)	13
rrm3Δ	1.0 (1.1)	3.87×10^{-8} (2.0)	24
siz1Δ	1.0	6.35×10^{-8} (3.2)	24

slx1Δ	1.0	2.32×10^{-8} (1.2)	13
slx4Δ	1.0 (1.1)	9.26×10^{-8} (4.7)	13
est1Δ	0.9	1.96×10^{-8} (1.0)	20
mrc1-aq	0.9 (1.0)	1.23×10^{-7} (6.2)	13
hcs1Δ	0.8	1.22×10^{-7} (6.2)	24
mgs1Δ	0.8	2.45×10^{-8} (1.2)	24
nhp10Δ	0.7	3.01×10^{-8} (1.5)	13
rad9Δ	0.5 (0.6)	3.82×10^{-8} (1.9)	13
taf14Δ/anc1Δ	0.0	2.02×10^{-8} (1.0)	24
arp8Δ	- (0.7)	4.84×10^{-8} (2.5)	13

*Scores in parentheses correspond to GCR strain scores from an *yfg*Δ *leu2*Δ double mutant cross, where *yfg*Δ ("*your favorite gene*") is the mutation listed in the first column.

Supplementary Table 4. Single mutant GCR rates determined for systematically generated tyGCR strains.

genotype	***GCR strain score***	***Can^R 5FOA^R rate****
*leu2*Δ	2.6	3.21[2.58-3.45] $\times 10^{-7}$ (1)
*rtt109*Δ	4.0	7.57 [5.90-12.2] $\times 10^{-6}$ (23.6)
nup84Δ	3.0	7.31 [5.32-12.2] $\times 10^{-6}$ (22.8)
*tsa1*Δ	4.0	6.61 [4.25-26.7] $\times 10^{-6}$ (20.6)
*dia2*Δ	4.3	5.81 [4.87-7.36] $\times 10^{-6}$ (18.1)
*slx5*Δ	3.7	5.69 [3.21-8.40] $\times 10^{-6}$ (17.7)
*ssz1*Δ	4.0	5.00 [4.46-7.63] $\times 10^{-6}$ (15.6)
*ctf4*Δ	4.0	2.69 [1.14-3.62] $\times 10^{-6}$ (8.4)
*clb5*Δ	4.0	2.45 [1.84-3.27] $\times 10^{-6}$ (7.6)
*vid22*Δ	3.3	2.45 [1.90-3.58] $\times 10^{-6}$ (7.6)
*srs2*Δ	4.0	1.97 [1.58-2.33] $\times 10^{-6}$ (6.1)
*rad51*Δ	4.0	1.60 [1.20-1.75] $\times 10^{-6}$ (5.0)
*ydj1*Δ	3.0	1.51 [0.97-2.65] $\times 10^{-6}$ (4.7)
*csm3*Δ	4.0	1.34 [0.95-1.52] $\times 10^{-6}$ (4.2)
*prr1*Δ	1.7	1.36 [1.06-1.39] $\times 10^{-6}$ (4.2)
*skn7*Δ	2.0	1.23 [1.01-1.84] $\times 10^{-6}$ (3.8)
*pol32*Δ	3.7	1.08 [0.64-1.90] $\times 10^{-6}$ (3.4)
*hst4*Δ	4.0	9.51 [6.99-10.4] $\times 10^{-7}$ (3.0)
*pfd1*Δ	1.3	9.16 [5.92-13.3] $\times 10^{-7}$ (2.9)
*msh2*Δ	2.3	8.78 [5.21-24.7] $\times 10^{-7}$ (2.7)
*cln3*Δ	3.0	6.70 [4.90-8.79] $\times 10^{-7}$ (2.1)
sae2Δ	3.0	6.66 [6.23-7.53] $\times 10^{-7}$ (2.1)
*chd1*Δ	3.7	6.40 [5.46-7.33] $\times 10^{-7}$ (2.0)
*rad9*Δ	3.0	5.25 [4.69-5.91] $\times 10^{-7}$ (1.6)
*rad10*Δ	3.0	4.95 [4.55-5.12] $\times 10^{-7}$ (1.5)
exo1Δ	3.0	4.41 [3.74-5.69] $\times 10^{-7}$ (1.4)
*mrs4*Δ	3.3	4.35 [3.49-5.69] $\times 10^{-7}$ (1.4)
*met18*Δ	3.7	4.57 [2.31-7.74] $\times 10^{-7}$ (1.4)
*esc1*Δ	3.3	4.19 [3.74-5.69] $\times 10^{-7}$ (1.3)
*slx1*Δ	2.0	2.75 [2.55-3.81] $\times 10^{-7}$ (0.9)
*poc4*Δ	1.7	1.22 [0.82-1.34] $\times 10^{-7}$ (0.4)
*rad52*Δ	1.7	8.31 [5.62-20.3] $\times 10^{-8}$ (0.3)
*nup133*Δ	0.3	5.56 [0.00-19.7] $\times 10^{-10}$ (0.0)

*Brackets are 95% confidence intervals and parentheses are fold increase over the *leu2*Δ control strain.

Supplementary Table 5. Single mutant GCR rates determined for systematically generated sGCR strains.

genotype	*GCR strain score*	*Can^R 5FOA^R rate**
leu2Δ	0.1	4.00 [2.43-7.48] $\times 10^{-9}$ (1)
ssz1Δ	1.0	1.90 [1.50-2.22] $\times 10^{-7}$ (47.5)
slx5Δ	0.0	1.56 [0.35-10.5] $\times 10^{-7}$ (39.0)
rad51Δ	1.0	9.26 [4.83-16.0] $\times 10^{-8}$ (23.2)
tsa1Δ	0.3	6.74 [3.46-8.65] $\times 10^{-8}$ (16.9)
sae2Δ	1.3	5.17 [3.61-6.02] $\times 10^{-8}$ (12.9)
rtt109Δ	0.3	5.01 [2.96-6.81] $\times 10^{-8}$ (12.5)
nup84Δ	0.0	3.99 [2.93-4.90] $\times 10^{-8}$ (10.0)
dia2Δ	0.0	3.11 [1.26-5.83] $\times 10^{-8}$ (7.8)
skn7Δ	0.3	2.50 [1.66-3.51] $\times 10^{-8}$ (6.3)
ydj1Δ	0.3	2.47 [1.84-3.62] $\times 10^{-8}$ (6.2)
vid22Δ	0.0	2.80 [2.57-8.77] $\times 10^{-8}$ (5.2)
met18Δ	0.3	1.57 [0.84-3.29] $\times 10^{-8}$ (3.9)
clb5Δ	0.0	1.26 [0.84-4.51] $\times 10^{-8}$ (3.5)
ctf4Δ	0.3	1.37 [0.33-2.15] $\times 10^{-8}$ (3.4)
exo1Δ	0.3	9.30 [6.30-20.5] $\times 10^{-9}$ (2.3)
rad9Δ	1.0	7.17 [3.89-7.95] $\times 10^{-9}$ (2.0)
rad52Δ	0.2	7.00 [2.91-3.07] $\times 10^{-9}$ (1.8)
csm3Δ	0.3	6.74 [5.61-13.7] $\times 10^{-9}$ (1.7)
cln3Δ	0.0	5.69 [3.53-17.8] $\times 10^{-9}$ (1.4)
hst4Δ	0.0	4.98 [2.19-7.12] $\times 10^{-9}$ (1.2)
pol32Δ	0.3	4.92 [2.08-10.4] $\times 10^{-9}$ (1.2)
esc1Δ	0.0	4.24 [2.14-6.52] $\times 10^{-9}$ (1.1)
chd1Δ	0.3	3.90 [2.12-1.57] $\times 10^{-9}$ (1.0)
poc4Δ	1.0	3.35 [1.88-9.84] $\times 10^{-9}$ (0.8)
mrs4Δ	0.0	2.27 [0.62-3.69] $\times 10^{-9}$ (0.6)
prr1Δ	0.0	2.42 [1.47-2.90] $\times 10^{-9}$ (0.6)
msh2Δ	0.0	2.13 [1.62-3.30] $\times 10^{-9}$ (0.5)
nup133Δ	0.0	2.05 [0.84-6.60] $\times 10^{-9}$ (0.5)
rad10Δ	1.0	2.00 [0.60-4.77] $\times 10^{-9}$ (0.5)
slx1Δ	1.0	1.81 [1.62-4.16] $\times 10^{-9}$ (0.5)
pfd1Δ	1.0	1.64 [0.00-4.48] $\times 10^{-9}$ (0.4)
srs2Δ	0.0	1.10 [0.74-3.67] $\times 10^{-9}$ (0.3)

*Brackets are 95% confidence intervals and parentheses are fold increase over the *leu2Δ* control strain.

Supplementary Table 6. Double mutant dGCR rates.

query mutation (dGCR strain score)	bait mutation (dGCR strain score)	double mutant GCR strain score	query mutation dGCR rate	bait mutation dGCR rate	double mutant dGCR rate
*mrc1*Δ (2.1)	*elg1*Δ (2.2)	5.0	3.08×10^{-7} (3.6)	2.42×10^{-7} (2.8)	2.49×10^{-6} (29)
*rsc30*Δ (0.3)	*lif1*Δ (1.3)	5.0	2.45×10^{-7} (2.9)	9.92×10^{-8} (1.2)	1.58×10^{-5} (184)
*sic1*Δ (2.4)	*hur1*Δ (0.8)	5.0	8.99×10^{-7} (10)	3.74×10^{-8} (0.4)	4.49×10^{-6} (52)
*swr1*Δ (1.3)	*eaf7*Δ (0.3)	5.0	1.32×10^{-7} (1.5)	9.93×10^{-8} (1.2)	4.47×10^{-6} (52)
*rsc30*Δ (0.3)	*lsm7*Δ (0.5)	4.7	2.45×10^{-7} (2.9)	5.86×10^{-8} (0.7)	5.68×10^{-6} (66)
*rrm3*Δ (1.1)	*nup133*Δ (2.0)	4.3	$1.02\text{x}10^{-7}$ (1.2)	1.21×10^{-6} (14)	3.46×10^{-6} (40)
*dun1*Δ (2.1)	*rnh201*Δ (1.2)	4.0	1.59×10^{-7} (1.9)	2.86×10^{-7} (3.3)	4.38×10^{-7} (5.1)
*dun1*Δ (2.1)	*rnh202*Δ (1.3)	4.0	1.59×10^{-7} (1.9)	1.85×10^{-7} (2.2)	4.42×10^{-7} (5.2)
*exo1*Δ (1.3)	*cdc73*Δ (1.7)	4.0	4.31×10^{-7} (5.0)	3.71×10^{-7} (4.3)	4.12×10^{-6} (48)
*exo1*Δ (1.3)	*chk1*Δ (1.4)	4.0	4.31×10^{-7} (5.0)	5.76×10^{-7} (6.7)	4.52×10^{-6} (53)
*exo1*Δ (1.3)	*ddc2*Δ *sml1*Δ (2.0)	4.0	4.31×10^{-7} (5.0)	2.39×10^{-7} (2.8)	9.02×10^{-6} (105)
*exo1*Δ (1.3)	*mec1*Δ *sml1*Δ (1.9)	4.0	4.31×10^{-7} (5.0)	5.57×10^{-7} (6.5)	1.12×10^{-5} (130)
*mph1*Δ (1.5)	*dun1*Δ (2.1)	4.0	2.96×10^{-7} (3.5)	1.59×10^{-7} (1.9)	1.46×10^{-6} (17)
*mrc1*Δ (2.1)	*dot1*Δ (0.3)	4.0	3.08×10^{-7} (3.6)	$7.10\text{x}10^{-8}$ (0.8)	2.49×10^{-6} (29)
*mrc1*Δ (2.1)	*slx4*Δ (1.1)	4.0	3.08×10^{-7} (3.6)	1.03×10^{-7} (1.2)	2.35×10^{-7} (2.7)
*mrc1*Δ (2.1)	*swr1*Δ (1.3)	4.0	3.08×10^{-7} (3.6)	1.32×10^{-7} (1.7)	3.96×10^{-6} (46)
*mrc1*Δ (2.1)	*vps72*Δ (1.1)	4.0	3.08×10^{-7} (3.6)	2.08×10^{-7} (2.4)	4.10×10^{-6} (48)
*pol32*Δ (1.3)	*msh6*Δ (1.8)	4.0	2.12×10^{-7} (2.5)	n.d.	3.94×10^{-6} (46)
srs2Δ (2.1)	*rnh201*Δ (1.2)	4.0	5.10×10^{-7} (5.9)	2.86×10^{-7} (3.3)	1.79×10^{-6} (21)
srs2Δ (2.1)	*rnh202*Δ (1.3)	4.0	5.10×10^{-7} (5.9)	1.85×10^{-7} (2.2)	2.28×10^{-6} (26)
*swr1*Δ (1.3)	*mrc1*Δ (2.1)	4.0	1.32×10^{-7} (1.5)	3.08×10^{-7} (3.6)	2.07×10^{-6} (24)
*dun1*Δ (2.1)	*yku70*Δ (1.3)	3.7	1.59×10^{-7} (1.9)	6.70×10^{-8} (0.8)	2.88×10^{-7} (3.4)
*lge1*Δ (1.0)	*mrc1*Δ (2.1)	3.7	1.12×10^{-7} (1.3)	3.08×10^{-7} (3.6)	1.34×10^{-6} (16)
*mph1*Δ (1.5)	*elg1*Δ (2.2)	3.7	2.96×10^{-7} (3.5)	2.42×10^{-7} (2.8)	1.35×10^{-6} (16)
*mrc1*Δ (2.1)	*rad51*Δ (1.3)	3.7	3.08×10^{-7} (3.6)	2.27×10^{-7} (2.6)	5.17×10^{-7} (6.0)
srs2Δ (2.1)	*rnh203*Δ (1.6)	3.7	5.10×10^{-7} (5.9)	1.73×10^{-7} (2.0)	2.00×10^{-6} (23)
*rad17*Δ (2.2)	*tel1*Δ (1.2)	3.3	5.70×10^{-7} (6.6)	3.38×10^{-7} (3.9)	9.96×10^{-7} (12)
*ckb2*Δ (1.3)	*chk1*Δ (1.4)	3.3	7.47×10^{-7} (8.7)	5.76×10^{-7} (6.7)	1.42×10^{-6} (16)
*ckb2*Δ (1.3)	*mec1*Δ *sml1*Δ (1.9)	3.3	7.47×10^{-7} (8.7)	5.57×10^{-7} (6.5)	9.09×10^{-7} (11)
*ckb2*Δ (1.3)	*mec3*Δ (0.8)	3.3	7.47×10^{-7} (8.7)	2.24×10^{-8} (0.3)	1.92×10^{-6} (22)
*ckb2*Δ (1.3)	*mrc1*Δ (2.1)	3.3	7.47×10^{-7} (8.7)	3.08×10^{-7} (3.6)	2.37×10^{-6} (28)
*ckb2*Δ (1.3)	*rad17*Δ (2.2)	3.3	7.47×10^{-7} (8.7)	5.70×10^{-7} (6.6)	4.01×10^{-6} (47)
*ckb2*Δ (1.3)	*rad24*Δ (2.1)	3.3	7.47×10^{-7} (8.7)	5.36×10^{-7} (6.2)	2.54×10^{-6} (30)
*mph1*Δ (1.5)	*cdc73*Δ (1.7)	3.3	2.96×10^{-7} (3.5)	3.71×10^{-7} (4.3)	1.30×10^{-6} (15)
*rsc30*Δ (0.3)	*csm3*Δ (1.8)	3.3	2.45×10^{-7} (2.9)	3.18×10^{-7} (3.3)	7.93×10^{-7} (9.2)
*ctf18*Δ (1.3)	*ckb1*Δ (1.0)	3.0	1.41×10^{-7} (1.6)	2.14×10^{-7} (2.5)	3.21×10^{-7} (3.7)
*exo1*Δ (1.3)	*ddc1*Δ (2.0)	3.0	4.31×10^{-7} (5.0)	3.58×10^{-7} (4.2)	2.27×10^{-6} (26)
*exo1*Δ (1.3)	*dun1*Δ (2.1)	3.0	4.31×10^{-7} (5.0)	1.59×10^{-7} (1.9)	3.05×10^{-6} (36)
*exo1*Δ (1.3)	*elg1*Δ (2.2)	3.0	4.31×10^{-7} (5.0)	2.42×10^{-7} (2.8)	1.36×10^{-6} (15.8)
*exo1*Δ (1.3)	*hos2*Δ (1.0)	3.0	4.31×10^{-7} (5.0)	1.02×10^{-7} (1.2)	3.13×10^{-7} (3.6)
*exo1*Δ (1.3)	*mec3*Δ (0.8)	3.0	4.31×10^{-7} (5.0)	2.24×10^{-8} (0.3)	9.92×10^{-7} (12)
*exo1*Δ (1.3)	*mrc1*Δ (2.1)	3.0	4.31×10^{-7} (5.0)	3.08×10^{-7} (3.6)	1.69×10^{-6} (20)
*mec1*Δ *sml1*Δ (1.9)	*ckb2*Δ (1.3)	3.0	7.47×10^{-7} (8.7)	7.47×10^{-7} (8.7)	1.44×10^{-7} (1.7)
*mec1*Δ *sml1*Δ (1.9)	*rnh202*Δ (1.3)	3.0	7.47×10^{-7} (8.7)	1.85×10^{-7} (2.2)	6.93×10^{-7} (8.1)
*mec1*Δ *sml1*Δ (1.9)	*spt8*Δ (1.4)	3.0	7.47×10^{-7} (8.7)	7.30×10^{-7} (8.5)	8.39×10^{-7} (9.8)
*pol32*Δ (1.3)	*msh2*Δ (2.2)	3.0	2.12×10^{-7} (2.5)	5.76×10^{-7} (6.7)	2.84×10^{-6} (33)

*rad17*Δ (2.2)	*rnh202*Δ (1.3)	3.0	5.70×10^{-7} (6.6)	1.85×10^{-7} (2.2)	7.49×10^{-7} (8.7)
*rad17*Δ (2.2)	*spt8*Δ (1.4)	3.0	5.70×10^{-7} (6.6)	7.30×10^{-7} (8.5)	6.45×10^{-7} (7.5)
*rad17*Δ (2.2)	*yku70*Δ (1.3)	3.0	5.70×10^{-7} (6.6)	6.70×10^{-8} (0.8)	1.75×10^{-6} (20)
*rev1*Δ (1.0)	*mph1*Δ (1.5)	3.0	4.19×10^{-7} (4.9)	2.96×10^{-7} (3.5)	8.11×10^{-6} (94)
*rev3*Δ (1.2)	*mph1*Δ (1.5)	3.0	3.29×10^{-7} (3.8)	2.96×10^{-7} (3.5)	7.00×10^{-6} (81.5)
*rtt107*Δ (1.7)	*cdh1*Δ (0.2)	3.0	4.95×10^{-7} (5.8)	7.80×10^{-8} (0.9)	4.22×10^{-7} (4.9)
*swr1*Δ (1.3)	*slx8*Δ (2.7)	3.0	1.32×10^{-7} (1.5)	7.30×10^{-6} (58)	1.18×10^{-6} (14)
*ckb2*Δ (1.3)	*csm3*Δ (1.8)	2.7	7.47×10^{-7} (8.7)	3.18×10^{-7} (3.3)	2.09×10^{-6} (24)
*exo1*Δ (1.3)	*tel1*Δ (1.2)	2.7	4.31×10^{-7} (5.0)	3.38×10^{-7} (3.9)	6.13×10^{-7} (7.1)
*rev3*Δ (1.2)	*psy3*Δ (0.8)	2.3	3.29×10^{-7} (3.8)	1.55×10^{-7} (1.8)	1.67×10^{-6} (19.4)
*rev3*Δ (1.2)	*shu1*Δ (1.1)	2.3	3.29×10^{-7} (3.8)	3.98×10^{-7} (4.6)	2.01×10^{-6} (23.4)
*lge1*Δ (1.0)	*exo1*Δ (1.3)	2.0	1.12×10^{-7} (1.3)	4.31×10^{-7} (5.0)	1.44×10^{-7} (1.7)
*mec1*Δ *sml1*Δ (1.9)	*yku70*Δ (1.3)	2.0	7.47×10^{-7} (8.7)	6.70×10^{-8} (0.8)	3.52×10^{-7} (4.1)
*mrc1*Δ (2.1)	*tel1*Δ (1.2)	2.0	3.08×10^{-7} (3.6)	3.38×10^{-7} (3.9)	2.87×10^{-7} (3.3)
*rev1*Δ (1.0)	*csm2*Δ (0.7)	2.0	4.19×10^{-7} (4.9)	8.29×10^{-7} (9.7)	1.65×10^{-6} (19)
*rev1*Δ (1.0)	*psy3*Δ (0.8)	2.0	4.19×10^{-7} (4.9)	1.55×10^{-7} (1.8)	1.50×10^{-6} (17)
*rev1*Δ (1.0)	*shu1*Δ (1.1)	2.0	4.19×10^{-7} (4.9)	3.98×10^{-7} (4.6)	2.26×10^{-6} (26)
*rev3*Δ (1.2)	*csm2*Δ (0.7)	2.0	3.29×10^{-7} (3.8)	8.29×10^{-7} (9.7)	1.59×10^{-6} (18.5)
*ckb2*Δ (1.3)	*mrc1-aq* (1.0)	1.7	7.47×10^{-7} (8.7)	1.01×10^{-7} (1.2)	3.21×10^{-7} (4.0)
*exo1*Δ (1.3)	*mrc1-aq* (1.0)	1.7	4.31×10^{-7} (5.0)	1.01×10^{-7} (1.2)	7.12×10^{-7} (8.3)
*exo1*Δ (1.3)	*rad9*Δ (0.7)	1.7	4.31×10^{-7} (5.0)	3.34×10^{-8} (3.9)	5.91×10^{-7} (6.9)
*rev1*Δ (1.0)	*shu2*Δ (1.3)	1.7	4.19×10^{-7} (4.9)	2.68×10^{-7} (3.1)	1.35×10^{-6} (16)
*rev3*Δ (1.2)	*shu2*Δ (1.3)	1.7	3.29×10^{-7} (3.8)	2.68×10^{-7} (3.1)	1.89×10^{-6} (22.0)
*ckb2*Δ (1.3)	*rad9*Δ (0.7)	1.3	7.47×10^{-7} (8.7)	3.34×10^{-8} (3.9)	4.53×10^{-7} (5.6)
*exo1*Δ (1.3)	*rad51*Δ (1.3)	1.3	4.31×10^{-7} (5.0)	2.27×10^{-7} (2.6)	1.65×10^{-7} (1.9)
*exo1*Δ (1.3)	*rad53*Δ *sml1*Δ (1.1)	1.3	4.31×10^{-7} (5.0)	2.48×10^{-7} (2.9)	2.56×10^{-7} (3.0)
*exo1*Δ (1.3)	*pol32*Δ (1.3)	0.7	4.31×10^{-7} (5.0)	2.12×10^{-7} (2.5)	3.50×10^{-6} (41)
*lge1*Δ (1.0)	*mre11*Δ (4.0)	0.7	1.12×10^{-7} (1.3)	2.88×10^{-6} (34)	9.42×10^{-8} (1.1)
*exo1*Δ (1.3)	*rad54*Δ (0.8)	0.3	4.31×10^{-7} (5.0)	1.31×10^{-7} (1.5)	6.60×10^{-8} (0.8)

Supplementary Table 7. Interactions of *ckb2*Δ and *exo1*Δ mutations with checkpoint defects.

	wild-type		*ckb2Δ*		*exo1Δ*	
	Rate*	GCR strain score	Rate*	GCR strain score	Rate*	GCR strain score
*leu2*Δ	8.59×10^{-8} (1)	0.95	7.47×10^{-7} (8.7)	1.3	4.31×10^{-7} (5.0)	1.3
*chk1*Δ	5.76×10^{-7} (6.7)	1.4	1.42×10^{-6} (16.5)	3.3	2.14×10^{-6} (24.9)	4.0
*csm3*Δ	3.18×10^{-7} (3.7)	1.8	2.09×10^{-6} (24.3)	2.7	n.d.	3.0
*ddc1*Δ	3.58×10^{-7} (4.2)	2.0	n.d.	3.3	2.27×10^{-6} (26.4)	3.0
*ddc2*Δ *sml1*Δ	2.39×10^{-7} (2.8)	2.0	n.d.	3.3	9.02×10^{-6} (105)	4.0
*dun1*Δ	1.59×10^{-7} (1.9)	2.1	n.d.	1.7	3.05×10^{-6} (35.6)	3.0
*elg1*Δ	2.42×10^{-7} (2.8)	2.2	n.d.	2.0	1.36×10^{-6} (15.8)	3.0
*mec1*Δ *sml1*Δ	5.57×10^{-7} (6.5)	1.9	9.09×10^{-7} (10.6)	3.3	1.06×10^{-5} (123)	4.0
*mec3*Δ	2.24×10^{-8} (0.3)	0.8	1.92×10^{-6} (22.4)	3.3	9.92×10^{-7} (11.5)	3.0
*mrc1*Δ	3.08×10^{-7} (3.6)	2.1	2.37×10^{-6} (27.6)	3.3	1.69×10^{-6} (19.7)	3.0
mrc1-aq	1.01×10^{-7} (1.2)	1.0	3.21×10^{-7} (4.0)	1.7	7.12×10^{-7} (8.3)	1.7
*rad9*Δ	3.34×10^{-8} (3.9)	0.7	4.53×10^{-7} (5.6)	1.3	5.91×10^{-7} (6.9)	1.7
*rad17*Δ	5.70×10^{-7} (6.6)	2.2	4.01×10^{-6} (46.7)	3.3	n.d.	3.0
*rad24*Δ	5.36×10^{-7} (6.2)	2.1	2.54×10^{-6} (29.6)	3.3	n.d.	3.0
*rad53*Δ *sml1*Δ	2.48×10^{-7} (2.9)	1.1	n.d.	1.3	2.56×10^{-7} (3.0)	1.3
*tel1*Δ	3.38×10^{-7} (3.9)	1.2	n.d.	2.0	6.13×10^{-7} (7.1)	2.7
*tof1*Δ		1.5		2.7		3.0
*ckb1*Δ	2.14×10^{-7} (2.5)	1.0	n.d.	2.0	n.d.	2.0
*ckb2*Δ	7.47×10^{-7} (8.7)	1.3	-	-	n.d.	2.7
*exo1*Δ	4.31×10^{-7} (5.0)	1.3	n.d.	2.3	-	-

*Rate of Can^R $5FOA^R$ colonies, fold increase over the *leu2*Δ control strain in parentheses. n.d. = not determined.

Supplementary Table 8. Summary of occurrence of mutations, reduced copy number and expression and predicted silencing of human GIS genes in ovarian and colorectal cancer.

Summary of Mutation Occurrence in GIS Genes - All Missense				
	Ovarian Cancer		Colorectal Cancer	
	nonsense, deletion, insertion, frameshift, splice site	nonsense, deletion, insertion, frameshift, splice site + missense	nonsense, deletion, insertion, frameshift, splice site	nonsense, deletion, insertion, frameshift, splice site + missense
# of samples with data	476	476	537	537
# of mutations	149	732	649	2700
# of samples with mutations	129	323	157	350
Range: mutation/ mutated sample	1 - 3	1 - 8	1 - 36	1 - 190
Range: mutation/ gene	1 - 55	1 - 195	1 - 32	1 - 87
Average # of mutations/mutated sample	1.2	2.3	4.1	7.7
# of genes with mutations	44	170	185	260

Summary of Mutation Occurrence in GIS Genes - Predicted Deleterious Missense				
	Ovarian Cancer		Colorectal Cancer	
	nonsense, deletion, insertion, frameshift, splice site	nonsense, deletion, insertion, frameshift, splice site + missense (Ndamage = 5, 6)	nonsense, deletion, insertion, frameshift, splice site	nonsense, deletion, insertion, frameshift, splice site + missense (Ndamage = 5, 6)
# of samples with data	476	476	537	537
# of mutations	149	221	649	1146
# of samples with mutations	129	176	157	221
Range: mutation/ mutated sample	1 - 3	1 -3	1 - 36	1 - 78
Range: mutation/ gene	1 - 55	1 - 57	1 - 32	1 - 47
Average # of mutations/mutated sample	1.2	1.3	4.1	5.2
# of genes with mutations	44	85	185	224

Summary of Reduced Copy Number/Expression of GIS Genes		
	Ovarian Cancer	Colorectal Cancer
	Reduced copy number (GISTIC -1, -2) and reduced expression (Zexp <= -2) [GISTIC -2 only]	Reduced copy number (GISTIC -1, -2) and reduced expression (Zexp <= -2) [GISTIC -2 only]
# of samples with data	527	456
# of alterations	3003 [200]	608 [20]
# of affected samples	508 [83]	246 [17]
Range: altered genes/affected sample	1 - 19 [1 - 9]	1 - 8 [1 - 2]
Range: alterations per gene	3 - 198 [1 - 16]	1 - 69 [1 - 5]
Average # of altered genes/affected sample	5.9 [2.4]	2.5 [1.2]
# of altered genes	45 [41]	20 [10]

Summary of Silenced GIS Genes		
	Ovarian Cancer	Colorectal Cancer
	Increased methylation (MetGL1 >= 0.5) and reduced expression (Zexp <= -1)	Increased methylation (MetGL1 >= 0.5) and reduced expression (Zexp <= -1)
# of samples with data	537	463
# of cases of silenced genes	63	58
# of affected samples	62	47
Range: silenced genes/affected sample	1 - 2	1 - 3
Range: alterations per gene	6 - 49	2 - 27
Average # of silenced genes/affected sample	1	1.2
# of genes showing silencing	3	4

Summary of Mutation Occurrence + Reduced Copy Number/Expression + Silencing in GIS Genes				
	Ovarian Cancer		Colorectal Cancer	
	nonsense, deletion, insertion, frameshift, splice site (samples with complete data)	nonsense, deletion, insertion, frameshift, splice site + missense Ndamage = 5, 6 (samples with complete data)	nonsense, deletion, insertion, frameshift, splice site (samples with complete data)	nonsense, deletion, insertion, frameshift, splice site + missense Ndamage = 5, 6 (samples with complete data)
# of samples with data	561 (452)	561 (452)	568 (426)	568 (426)
# of samples with mutations only	9	11	103	133
# of samples with reduced copy number and expression only	337	308	191	164
# of samples with silenced genes only	2	1	9	5
# of samples with mutations and reduced copy number and expression	111	140	34	61
# of samples with mutations and silenced genes	0	1	17	21
# of samples with reduced copy number and expression and silenced genes	52	46	18	15
# of samples with all three classes of alterations	8	14	3	6
# of samples with an alteration	519	521	375	405
Range: alterations/affected sample	1 - 20	1 - 20	1 - 36	1 - 78
Range: alterations/gene	1 - 198	1 - 199	1 - 70	1 - 72
Average # of alterations/affected sample	6.3	6.4	3.5	4.5

Supplementary Table 9. *S. cerevisiae* strains used in this study.

Name	Genotype
RDKY7635	MATα *hom3-10 ura3Δ0 leu2Δ0 trp1Δ63 his3Δ200 lyp1::TRP1 cyh2-Q38K iYFR016C::P$_{M-FA1}$-LEU2 can1::P$_{LEU2}$-NAT yel072w::CAN1/URA3*
RDKY7712	RDKY7635 *arp8::HIS3*
RDKY8042	RDKY8042 *ckb2::HIS3*
RDKY7714	RDKY7635 *ctf18::HIS3*
RDKY7779	RDKY7635 *ctk2::HIS3*
RDKY7715	RDKY7635 *dia2::HIS3*
RDKY7784	RDKY7635 *dun1::HIS3*
RDKY7717	RDKY7635 *elg1::HIS3*
RDKY7638	RDKY7635 *exo1::HIS3*
RDKY7644	RDKY7635 *fun30::HIS3*
RDKY8034	RDKY7635 *hos2::HIS3*
RDKY7642	RDKY7635 *lge1::HIS3*
RDKY8045	RDKY7635 *mec1::HIS3 sml1::hph*
RDKY7780	RDKY7635 *mms4::HIS3*
RDKY7744	RDKY7635 *mph1::HIS3*
RDKY7636	RDKY7635 *mrc1::HIS3*
RDKY8044	RDKY7635 *mrc1-aq.HIS3*
RDKY7649	RDKY7635 *nup84::HIS3*
RDKY7725	RDKY7635 *pol32::HIS3*
RDKY7719	RDKY7635 *rad9::HIS3*
RDKY8049	RDKY7635 *rad17::HIS3*
RDKY7726	RDKY7635 *rad18::HIS3*
RDKY7736	RDKY7635 *rad51::HIS3*
RDKY7967	RDKY7635 *rad52::HIS3*
RDKY8047	RDKY7635 *rad53::HIS3 sml1::hph*
RDKY7738	RDKY7635 *rad59::HIS3*
RDKY7781	RDKY7635 *rdh54::HIS3*
RDKY7980	RDKY7635 *rev1::HIS3*
RDKY7983	RDKY7635 *rev3::HIS3*
RDKY7650	RDKY7635 *rrm3::HIS3*
RDKY7646	RDKY7635 *rsc30::HIS3*
RDKY7988	RDKY7635 *rtf1::HIS3*
RDKY7721	RDKY7635 *rtt101::HIS3*
RDKY7640	RDKY7635 *rtt107::HIS3*
RDKY7723	RDKY7635 *rtt109::HIS3*
RDKY8038	RDKY7635 *sem1::HIS3*
RDKY7782	RDKY7635 *sic1::HIS3*
RDKY7652	RDKY7635 *slx4::HIS3*
RDKY7783	RDKY7635 *slx8::HIS3*
RDKY7654	RDKY7635 *srs2::HIS3*
RDKY8036	RDKY7635 *spt8::HIS3*
RDKY7785	RDKY7635 *swr1::HIS3*
RDKY8040	RDKY7635 *yta7::HIS3*
RDKY7964	MATα *hom3-10 ura3Δ0 leu2Δ0 trp1Δ63 his3Δ200 lyp1::TRP1 cyh2-Q38K iYFR016C::P$_{M-FA1}$-LEU2 can1::P$_{LEU2}$-NAT yel068c::CAN1/URA*3

RDKY7967	RDKY7964 *rad52::HIS3*
RDKY7046	MATα *hom3-10 ura3Δ0 leu2Δ0 trp1Δ63 his3Δ200 lyp1::TRP1 cyh2-Q38K iYFR016C::*P_{MFA1}*-LEU2 iYEL062W::Ty912-hphNT1 hxt13::URA3*
BY404	MAT**a** *ade2::hisG his3Δ200 leu2Δ0 trp1Δ63 ura3Δ0*
RDKY3686	MATα *hom3-10 lys2-10A his3Δ200 leu2Δ1 trp1Δ63 ura3-52*
RDKY7594	MATα *lys2-10A hom3-10 his3Δ200 leu2Δ0 trp1Δ63 ura3Δ0*
RDKY7595	MAT**a** *lys2-10A hom3-10 his3Δ200 leu2Δ0 trp1Δ63 ura3Δ0*
RDKY7596	MAT**a** *lys2-10A hom3-10 his3Δ200 leu2Δ0 trp1Δ63 ura3Δ0 iYFR016C::URA3*
RDKY7597	MAT**a** *lys2-10A hom3-10 his3Δ200 leu2Δ0 trp1Δ63 ura3Δ0 iYFR016C::*P_{MFA1}*-LEU2*
RDKY7598	MATα *lys2-10A hom3-10 his3Δ200 leu2Δ0 trp1Δ63 ura3Δ0 iYFR016C::*P_{MFA1}*-LEU2*
RDKY7599	MATα *lys2-10A hom3-10 his3Δ200 leu2Δ0 trp1Δ63 ura3Δ0 iYFR016C::*P_{MFA1}*-LEU2 hxt13::URA3*
RDKY6970	MATα *hom3-10 his3Δ200 leu2Δ0 trp1Δ63 ura3Δ0 iYFR016C::*P_{MFA1}*-LEU2 hxt13::URA3*
RDKY6971	MATα *hom3-10 his3Δ200 leu2Δ0 trp1Δ63 ura3Δ0 iYFR016C::*P_{MFA1}*-LEU2 lyp1::TRP1 hxt13::URA3*
RDKY6975	MATα *hom3-10 his3Δ200 leu2Δ0 trp1Δ63 ura3Δ0 iYFR016C::*P_{MFA1}*-LEU2 lyp1::TRP1 cyh2-Q38K hxt13::URA3*
RDKY7625	MATα *hom3-10 his3Δ200 leu2Δ0 trp1Δ63 ura3Δ0 lyp1::TRP1 iYFR016C::*P_{MFA1}*-LEU2 cyh2-Q38K*
RDKY7629	MATα *hom3-10 his3Δ200 leu2Δ0 trp1Δ63 ura3Δ0 lyp1::TRP1 iYFR016C::*P_{MFA1}*-LEU2 cyh2-Q38K can1::*P_{LEU2}*-NAT*
RDKY6593	MAT**a** *lys2-10A hom3-10 ura3Δ0 leu2Δ0 trp1Δ63 his3Δ200 iYEL062W::Ty912-hphNT1 yel069c::URA3*

Supplementary Table 10. Strains constructed to replace or supplement those in the BY4741 deletion collection.

Rationale	***Gene***	***Marker***	***Note***
replace	*YBR194W*	*aim4::G418*	PCR-mediated gene disruption in BY4741
replace	*YJL115W*	*asf1::G418*	From BY4741xBY4742 *asf1::G418* cross
replace	*YGR188C*	*bub1::G418*	From BY4741xBY4742 *bub1::G418* cross
replace	*YAL021C*	*ccr4::G418*	From BY4741xBY4742 *ccr4::G418* cross
replace	*YCR002C*	*cdc10::G418*	PCR-mediated gene disruption in BY4741
replace	*YGL019W*	*ckb1::G418*	PCR-mediated gene disruption in BY4741
replace	*YMR078C*	*ctf18::G418*	From BY4741xBY4742 *ctf18::G418* cross
replace	*YKL139W*	*ctk1::G418*	From BY4741xBY4742 *ctk1::G418* cross
replace	*YML112W*	*ctk3::G418*	From BY4741xBY4742 *ctk3::G418* cross
replace	*YOL145C*	*ctr9::G418*	From BY4741xBY4742 *ctr9::G418* cross
replace	*YCL016C*	*dcc1::G418*	From BY4741xBY4742 *dcc1::G418* cross
replace	*YFL001W*	*deg1::G418*	PCR-mediated gene disruption in BY4741
replace	*YOR080W*	*dia2::G418*	From BY4741xBY4742 *dia2::G418* cross
replace	*YDR359C*	*eaf1::G418*	From BY4741xBY4742 *eaf1::G418* cross
replace	*YEL018W*	*eaf5::G418*	PCR-mediated gene disruption in BY4741
replace	*YJR082C*	*eaf6::G418*	From BY4741xBY4742 *eaf6::G418* cross
replace	*YOR033C*	*exo1::G418*	From BY4741xBY4742 *exo1::G418* cross
replace	*YOL051W*	*gal11:G418*	PCR-mediated gene disruption in BY4741
replace	*YJR090C*	*grr1::G418*	PCR-mediated gene disruption in BY4741
replace	*YOL095C*	*hmi1::G418*	From BY4741xBY4742 *hmi1::G418* cross
replace	*YGL168W*	*hur1::G418*	From BY4741xBY4742 *hur1::G418* cross
replace	*YLR384C*	*iki3::G418*	PCR-mediated gene disruption in BY4741
replace	*YDR332W*	*irc3::G418*	PCR-mediated gene disruption in BY4741
replace	*YDL115C*	*iwr1::G418*	PCR-mediated gene disruption in BY4741
replace	*YJL124C*	*lsm1::G418*	From BY4741xBY4742 *lsm1::G418* cross
replace	*YJL030W*	*mad2::G418*	From BY4741xBY4742 *mad2::G418* cross
replace	*YPR051W*	*mak3::G418*	From BY4741xBY4742 *mak3::G418* cross
replace	*YCR020C-A*	*mak31::G418*	PCR-mediated gene disruption in BY4741
replace	*YOL076W*	*mdm20::G418*	From BY4741xBY4742 *mdm20::G418* cross
replace	*YPR070W*	*med1::G418*	PCR-mediated gene disruption in BY4741
replace	*YDL040C*	*nat1::G418*	PCR-mediated gene disruption in BY4741
replace	*YAL015C*	*ntg1::G418*	From BY4741xBY4742 *ntg1::G418* cross
replace	*YAR002W*	*nup60::G418*	From BY4741xBY4742 *nup60::G418* cross
replace	*YDR113C*	*pds1::G418*	PCR-mediated gene disruption in BY4741
replace	*YML061C*	*pif1::G418*	From BY4741xBY4742 *pif1::G418* cross
replace	*YNR052C*	*pop2::G418*	From BY4741xBY4742 *pop2::G418* cross
replace	*YPL022W*	*rad1::G418*	From BY4742xBY4741 *rad1::G418* backcross; original BY4741 *rad1::G418* strain had HU- sensitivity that did not cosegregate with the *rad1::G418* marker
replace	*YLR176C*	*rfx1::G418*	From BY4741xBY4742 *rfx1::G418* cross

replace	*YNL139C*	*rlr1::G418*	From BY4741xBY4742 *rlr1::G418* cross
replace	*YIL066C*	*rnr3::G418*	From BY4741xBY4742 *rnr3::G418* cross
replace	*YNL330C*	*rpd3::G418*	From BY4741xBY4742 *rpd3::G418* cross
replace	*YHR200W*	*rpn10::G418*	From BY4741xBY4742 *rpn10::G418* cross
replace	*YHR056C*	*rsc30::G418*	PCR-mediated gene disruption in BY4741
replace	*YJL047C*	*rtt101::G418*	From BY4741xBY4742 *rtt101::G418* cross
replace	*YER104W*	*rtt105::G418*	PCR-mediated gene disruption in BY4741
replace	*YLL002W*	*rtt109::G418*	From BY4741xBY4742 *rtt109::G418* cross
replace	*YGL127C*	*soh1::G418*	From BY4741xBY4742 *soh1::G418* cross
replace	*YJL127C*	*spt10::G418*	From BY4741xBY4742 *spt10::G418* cross
replace	*YBR081C*	*spt7::G418*	PCR-mediated gene disruption in BY4741
replace	*YHR041C*	*srb2::G418*	PCR-mediated gene disruption in BY4741
replace	*YBR111W-A*	*sus1::G418*	PCR-mediated gene disruption in BY4741
replace	*YAL011W*	*swc3::G418*	From BY4741xBY4742 *swc3::G418* cross
replace	*YPL129W*	*taf14::G418*	From BY4741xBY4742 *taf14::G418* cross
replace	*YDR079C-A*	*tfb5::G418*	PCR-mediated gene disruption in BY4741
replace	*YOL072W*	*thp1::G418*	From BY4741xBY4742 *thp1::G418* cross
replace	*YEL012W*	*ubc8::G418*	From BY4741xBY4742 *ubc8::G418* cross
replace	*YFR010W*	*ubp6::G418*	PCR-mediated gene disruption in BY4741
replace	*YHR003C*	*yhr003c::G418*	From BY4741xBY4742 *yhr003c::G418* cross
replace	*YHR090C*	*yng2::G418*	PCR-mediated gene disruption in BY4741
replace	*YLR262C*	*ypt6::G418*	PCR-mediated gene disruption in BY4741
supplement	*YCL018W*	*leu2::G418*	PCR-mediated gene disruption in BY4741
supplement	*YDR499W*	*ddc2::G418 sml1::hph*	PCR-mediated gene disruption in BY4741
supplement	*YBR136W*	*mec1::G418 sml1::hph*	PCR-mediated gene disruption in BY4741
supplement	*YPL153C*	*rad53::G418 sml1::hph*	PCR-mediated gene disruption in BY4741
supplement	*YPL153C*	*rad53-21.G418*	PCR-mediated gene disruption in BY4741
supplement	*YCL061C*	*mrc1-aq.G418*	PCR-mediated gene replacement in BY4741

Supplementary References

1 Putnam, C. D., Hayes, T. K. & Kolodner, R. D. Specific pathways prevent duplication-mediated genome rearrangements. *Nature* **460**, 984-989, doi:10.1038/nature08217 (2009).

2 Putnam, C. D., Hayes, T. K. & Kolodner, R. D. Post-replication repair suppresses duplication-mediated genome instability. *PLoS Genetics* **6**, e1000933, doi:10.1371/journal.pgen.1000933 (2010).

3 Mazon, G. & Symington, L. S. Mph1 and Mus81-Mms4 prevent aberrant processing of mitotic recombination intermediates. *Molecular Cell* **52**, 63-74, doi:10.1016/j.molcel.2013.09.007 (2013).

4 Myung, K., Datta, A. & Kolodner, R. D. Suppression of spontaneous chromosomal rearrangements by S phase checkpoint functions in *Saccharomyces cerevisiae*. *Cell* **104**, 397-408 (2001).

5 Bando, M. *et al.* Csm3, Tof1, and Mrc1 form a heterotrimeric mediator complex that associates with DNA replication forks. *The Journal of Biological Chemistry* **284**, 34355-34365, doi:10.1074/jbc.M109.065730 (2009).
